# General iterative methods for systems of variational inequalities with the constraints of generalized mixed equilibria and fixed point problem of pseudocontractions

**DOI:** 10.1186/s13660-018-1899-0

**Published:** 2018-11-16

**Authors:** Qian-Wen Wang, Jin-Lin Guan, Lu-Chuan Ceng, Bing Hu

**Affiliations:** 1 Department of Mathematics, Shanghai Normal University, Shanghai, China; 2 LAMPS and Department of Mathematics and Statistics, York University, Toronto, Canada

**Keywords:** 47J20, 47H05, 47H09, 49J40, 49M05, General iterative method, General system of variational inequalities, Continuous monotone mapping, Continuous pseudocontractive mapping, Variational inequality, Generalized mixed equilibrium problem

## Abstract

In this paper, we introduce two general iterative methods (one implicit method and one explicit method) for finding a solution of a general system of variational inequalities (GSVI) with the constraints of finitely many generalized mixed equilibrium problems and a fixed point problem of a continuous pseudocontractive mapping in a Hilbert space. Then we establish strong convergence of the proposed implicit and explicit iterative methods to a solution of the GSVI with the above constraints, which is the unique solution of a certain variational inequality. The results presented in this paper improve, extend, and develop the corresponding results in the earlier and recent literature.

## Introduction

Let *C* be a nonempty closed convex subset of a real Hilbert space *H* with inner product $\langle \cdot , \cdot \rangle $ and induced norm $\Vert \cdot \Vert $. We denote by $P_{C}$ the metric projection of *H* onto *C* and by $\operatorname {Fix}(S)$ the set of fixed points of the mapping *S*. Recall that a mapping $T:C\to H$ is nonexpansive if $\Vert Tx-Ty\Vert \leq \Vert x-y \Vert $, $\forall x, y\in C$. A mapping $T: C\to H$ is called pseudocontractive if

$$ \langle Tx-Ty, x-y\rangle \leq \Vert x-y \Vert ^{2}, \quad \forall x, y\in C. $$

This inequality can be equivalently rewritten as

$$ \Vert Tx-Ty \Vert ^{2}\leq \Vert x-y \Vert ^{2}+ \bigl\Vert (I-T)x-(I-T)y \bigr\Vert ^{2}, \quad \forall x, y\in C, $$

where *I* is the identity mapping.

$T: C\to H$ is said to be *k*-strictly pseudocontractive if there exists a constant $k\in [0, 1)$ such that

$$ \Vert Tx-Ty \Vert ^{2}\leq \Vert x-y \Vert ^{2}+k \bigl\Vert (I-T)x-(I-T)y \bigr\Vert ^{2}, \quad \forall x, y\in C. $$

A mapping $V: C\to H$ is said to be *l*-Lipschitzian if there exists a constant $l\geq 0$ such that

$$ \Vert Vx-Vy \Vert \leq l \Vert x-y \Vert , \quad \forall x, y\in C. $$

A mapping $F: C\to H$ is called monotone if

$$ \langle x-y, Fx-Fy\rangle \geq 0, \quad \forall x, y\in C, $$

and *F* is called *α*-inverse-strongly monotone if there exists a constant $\alpha >0$ such that

$$ \langle x-y, Fx-Fy\rangle \geq \alpha \Vert Fx-Fy \Vert ^{2}, \quad \forall x, y\in C. $$

If *F* is an *α*-inverse-strongly monotone mapping, then it is obvious that *F* is $\frac{1}{\alpha }$-Lipschitz continuous, that is, $\Vert Fx-Fy\Vert \leq \frac{1}{\alpha }\Vert x-y\Vert $ for all $x, y\in C$.

A mapping $F: C\to H$ is called *β*-strongly monotone if there exists a constant $\beta >0$ such that

$$ \langle x-y, Fx-Fy\rangle \geq \beta \Vert x-y \Vert ^{2}, \quad \forall x, y \in C. $$

A linear operator $A: H\to H$ is said to be strongly positive on *H* if there exists a constant $\bar{\gamma }>0$ such that

$$ \langle Ax, x\rangle \geq \bar{\gamma } \Vert x \Vert ^{2}, \quad \forall x \in H. $$

Let $F: C\to H$ be a mapping. The classical variational inequality problem (VIP) is to find $x^{*}\in C$ such that

1.1 $$ \bigl\langle Fx^{*}, x-x^{*}\bigr\rangle \geq 0,\quad \forall x\in C. $$

We denote the set of solutions of VIP (1.1) by $\operatorname {VI}(C, F)$.

In 2008, Ceng et al. [1] considered the following general system of variational inequalities (GSVI):

1.2 $$ \textstyle\begin{cases} \langle \lambda F_{1}y^{*}+x^{*}-y^{*}, x-x^{*}\rangle \geq 0, \quad \forall x\in C, \\ \langle \nu F_{2}x^{*}+y^{*}-x^{*}, x-y^{*}\rangle \geq 0, \quad \forall x\in C, \end{cases} $$

where $F_{1}$, $F_{2}$ are *α*-inverse-strongly monotone and *β*-inverse-strongly monotone, respectively, and $\lambda \in (0, 2\alpha )$ and $\nu \in (0, 2\beta )$ are two constants. Many iterative methods have been developed for solving GSVI (1.2); see [2–7] and the references therein.

Subsequently, Alofi et al. [8] also introduced two composite iterative algorithms based on the composite iterative methods in Ceng et al. [9] and Jung [10] for solving the problem of GSVI (1.2). Moreover, they showed strong convergence of the proposed algorithms to a common solution of these two problems.

Very recently, Kong et al. [11] established the strong convergence of two hybrid steepest-descent schemes to the same solution of GSVI (1.2), which is also a common solution of finitely many variational inclusions and a minimization problem.

### Lemma 1.1

(see [12, Proposition 3.1])

*Let* *C* *be a nonempty closed convex subset of a real Hilbert space H*. *For given* $x^{*}, y^{*} \in C$, $(x^{*}, y^{*})$ *is a solution of GSVI* (1.3) *for continuous monotone mappings* $F_{1}$ *and* $F_{2}$ *if and only if* $x^{*}$ *is a fixed point of the composite* $R=F_{1, \lambda }F_{2, \nu }: H\to C$ *of nonexpansive mappings* $F_{1, \lambda }: H\to C$ *and* $F_{2,\nu }: H \to C$, *where* $y^{*}=F_{2, \nu }x^{*}$,

$$ F_{1, \lambda }x=\biggl\{ z\in C: \langle y-z, F_{1}z\rangle + \frac{1}{ \lambda }\langle y-z, z-x\rangle \geq 0, \forall y\in C\biggr\} , $$

*and*

$$ F_{2, \nu }x=\biggl\{ z\in C: \langle y-z, F_{2}z\rangle + \frac{1}{\nu } \langle y-z, z-x\rangle \geq 0, \forall y\in C\biggr\} . $$

*For simplicity*, *we denote by* $\operatorname {GSVI}(C,F_{1}, F_{2})$ *the fixed point set of mapping R*.

In the meantime, inspired by Ceng et al. [1], Jung [12] introduced a general system of variational inequalities (GSVI) for two continuous monotone mappings $F_{1}$ and $F_{2}$ of finding $(x^{*}, y^{*}) \in C\times C$ such that

1.3 $$ \textstyle\begin{cases} \langle \lambda F_{1}x^{*}+x^{*}-y^{*}, x-x^{*}\rangle \geq 0, \quad \forall x\in C, \\ \langle \nu F_{2}y^{*}+y^{*}-x^{*}, x-y^{*}\rangle \geq 0, \quad \forall x\in C, \end{cases} $$

where $\lambda , \nu >0$ are two constants. In order to find an element of $\operatorname {Fix}(R)\cap \operatorname {Fix}(T)$, he proposed one implicit algorithm generating a net $\{x_{t}\}$:

1.4 $$ x_{t}=(I-\theta_{t}A)T_{r_{t}}Rx_{t}+ \theta_{t}\bigl[t\gamma Vx_{t}+(I-t \mu G)T_{r_{t}}Rx_{t} \bigr], $$

with $t\in (0, \min \{1, \frac{2-\bar{\gamma }}{\tau -\gamma l}\})$ and $\theta_{t}\in (0, \min \{\frac{1}{2}, \Vert A\Vert ^{-1}\})$, and an explicit algorithm generating a sequence $\{x_{n}\}$:

1.5 $$ \textstyle\begin{cases} y_{n}=\alpha_{n}\gamma Vx_{n}+(I-\alpha_{n}\mu G)T_{r_{n}}Rx_{n}, \\ x_{n+1}=(I-\beta_{n}A)T_{r_{n}}Rx_{n}+\beta_{n}y_{n}, \quad \forall n \geq 0, \end{cases} $$

with $\{\alpha_{n}\}\subset [0,1]$, $\{\beta_{n}\}\subset (0,1]$, $\{r _{n}\}\subset (0,\infty )$, and $x_{0}\in C$ any initial guess, where $T_{r_{t}}x=\{z\in C: \langle y-z, Tz\rangle -\frac{1}{r_{t}}\langle y-z, (1+r_{t})z-x\rangle \leq 0, \forall y\in C\}$ for $r_{t}\in (0, \infty )$, and $T_{r_{n}}x=\{z\in C: \langle y-z, Tz\rangle -\frac{1}{r _{n}}\langle y-z, (1+r_{n})z-x \rangle \leq 0, \forall y\in C\}$ for $r_{n}\in (0,\infty )$. Moreover, he established strong convergence of the proposed iterative algorithms to an element $\widetilde{x}\in \operatorname {Fix}(R)\cap \operatorname {Fix}(T)$, which uniquely solves the variational inequality

$$ \bigl\langle (A-I)\widetilde{x}, \widetilde{x}-p\bigr\rangle \leq 0,\quad \forall p\in \operatorname {Fix}(R)\cap \operatorname {Fix}(T). $$

On the other hand, the generalized mixed equilibrium problem (GMEP) is to find $x\in C$ such that

1.6 $$ {{\varTheta }}(x, y)+\varphi (y)-\varphi (x)+\langle Bx, y-x\rangle \geq 0, \quad \forall y\in C. $$

We denote the set of solutions of GMEP (1.6) by $\operatorname {GMEP}({\varTheta }, \varphi , B)$. GMEP (1.6) is very general in the sense that it includes many problems as special cases, namely optimization problems, variational inequalities, minimax problems, Nash equilibrium problems in noncooperative games, and others. For different aspects and solution methods, we refer to [13–18] and the references therein.

In this paper, we introduce implicit and explicit iterative methods for finding a solution of GSVI (1.3) with solutions belonging also to the common solution set $\bigcap^{N}_{i=1}\operatorname {GMEP}({\varTheta }_{i}, \varphi_{i}, B_{i})$ of finitely many generalized mixed equilibrium problems and the fixed point set of a continuous pseudocontractive mapping *T*. First, GSVI (1.3) and each generalized mixed equilibrium problem both are transformed into fixed point problems of nonexpansive mappings. Then we establish strong convergence of the proposed iterative methods to an element of $\bigcap^{N}_{i=1}\operatorname {GMEP}({\varTheta } _{i}, \varphi_{i}, B_{i})\cap \operatorname {GSVI}(C, F_{1}, F_{2})\cap \operatorname {Fix}(T)$, which is the unique solution of a certain variational inequality.

## Preliminaries and lemmas

Let *H* be a real Hilbert space, and let *C* be a nonempty closed convex subset of *H*. We write $x_{n}\to x$ and $x_{n} \rightharpoonup x$ to indicate the strong convergence of the sequence $\{x_{n}\}$ to *x* and the weak convergence of the sequence $\{x_{n}\}$ to *x*, respectively.

For every point $x\in H$, there exists a unique nearest point in *C*, denoted by $P_{C}(x)$, such that

$$ \bigl\Vert x-P_{C}(x) \bigr\Vert \leq \Vert x-y \Vert , \quad \forall y\in C. $$

$P_{C}$ is called the metric projection of *H* onto *C*. It is well known that $P_{C}$ is nonexpansive and is characterized by the property

2.1 $$ u=P_{C}(x)\quad \Leftrightarrow \quad \langle x-u, u-y\rangle \geq 0, \quad \forall x\in H, y\in C. $$

In a Hilbert space *H*, the following equality holds:

2.2 $$ \Vert x-y \Vert ^{2}= \Vert x \Vert ^{2}+ \Vert y \Vert ^{2}-2\langle x, y\rangle , \quad \forall x, y\in H. $$

The following lemma is an immediate consequence of an inner product.

### Lemma 2.1

*In a real Hilbert space* *H*, *there holds the following inequality*:

$$ \Vert x+y \Vert ^{2}\leq \Vert x \Vert ^{2}+2\langle y, x+y\rangle ,\quad \forall x, y \in H. $$

Next we list some elementary conclusions for the MEP.

It is first assumed as in [19] that ${\varTheta }: C\times C\to {\mathbf{R}}$ is a bifunction satisfying conditions (A1)–(A4) and $\varphi : C\to {\mathbf{R}}$ is a lower semicontinuous and convex function with restriction (B1) or (B2), where

1. ${\varTheta }(x, x)=0$ for all $x\in C$;
2. *Θ* is monotone, i.e., ${\varTheta }(x, y)+{\varTheta }(y, x)\leq 0$ for any $x, y\in C$;
3. *Θ* is upper-hemicontinuous, i.e., for each $x, y, z\in C$,
  $$ \limsup_{t\to 0^{+}}{{\varTheta }}\bigl(tz+(1-t)x, y\bigr)\leq { {\varTheta }}(x, y); $$
4. ${\varTheta }(x, \cdot )$ is convex and lower semicontinuous for each $x\in C$;
5. for $\forall x\in H$ and $r>0$, there exists a bounded subset $D_{x}\subset C$ and $y_{x}\in C$ such that, for $\forall z\in C \setminus D_{x}$,
  $$ {{\varTheta }}(z, y_{x})+\varphi (y_{x})-\varphi (z)+ \frac{1}{r}\langle y_{x}-z, z-x\rangle < 0; $$
6. *C* is a bounded set.

### Proposition 2.1

([19])

*Assume that* ${\varTheta }: C\times C \to {\mathbf{R}}$ *satisfies* (A1)*–*(A4), *and let* $\varphi : C\to {\mathbf{R}}$ *be a proper lower semicontinuous and convex function*. *Assume that either* (B1) *or* (B2) *holds*. *For* $r>0$ *and* $x\in H$, *define a mapping* $T^{({\varTheta },\varphi )}_{r}: H\to C$ *as follows*:

$$ T^{({\varTheta },\varphi )}_{r}(x):=\biggl\{ z\in C:{\varTheta }(z, y)+ \varphi (y)-\varphi (z)+\frac{1}{r}\langle y-z, z-x\rangle \geq 0, \forall y\in C\biggr\} $$

*for all* $x\in H$. *Then the following hold*:

(i) *for each* $x\in H$, $T^{({\varTheta }, \varphi )}_{r}(x)$ *is nonempty and single*-*valued*;
(ii) $T^{({\varTheta },\varphi )}_{r}$ *is firmly nonexpansive*, *that is*, *for any* $x, y\in H$,
  $$ \bigl\Vert T^{({\varTheta }, \varphi )}_{r}x-T^{({\varTheta }, \varphi )} _{r}y \bigr\Vert ^{2}\leq \bigl\langle T^{({\varTheta }, \varphi )}_{r}x-T^{({\varTheta }, \varphi )}_{r}y, x-y\bigr\rangle ; $$
(iii) $\operatorname {Fix}(T^{({\varTheta }, \varphi )}_{r})=\operatorname {MEP}({\varTheta }, \varphi )$;
(iv) $\operatorname {MEP}({\varTheta }, \varphi )$ *is closed and convex*;
(v) $\Vert T^{({\varTheta },\varphi )}_{s}x-T^{({\varTheta }, \varphi )} _{t}x\Vert ^{2}\leq \frac{s-t}{s}\langle T^{({\varTheta },\varphi )}_{s}x-T ^{({\varTheta },\varphi )}_{t}x,T^{({\varTheta }, \varphi )}_{s}x-x \rangle $ *for all* $s, t>0$ *and* $x\in H$.

### Proposition 2.2

*Let* $F:C\to H$ *be an* *α*-*inverse*-*strongly monotone mapping*. *Then*, *for all* $x,y\in C$ *and* $\lambda >0$, *one has*

$$ \bigl\Vert (I-\lambda F)x-(I-\lambda F)y \bigr\Vert ^{2}\leq \Vert x-y \Vert ^{2}+\lambda (\lambda -2\alpha ) \Vert Fx-Fy \Vert ^{2}. $$

*In particular*, *if* $\lambda \in (0, 2\alpha ]$, $I-\lambda F: C\to H$ *is a nonexpansive mapping*.

We will use the following lemmas for the proof of our main results in the sequel.

### Lemma 2.2

([20])

*Let* $\{s_{n}\}$ *be a sequence of nonnegative real numbers satisfying*

$$ s_{n+1}\leq (1-\omega_{n})s_{n}+ \omega_{n}\delta_{n}+\gamma_{n}, \quad \forall n \geq 0, $$

*where* $\{\omega_{n}\}$, $\{\delta_{n}\}$, *and* $\{\gamma_{n}\}$ *satisfy the following conditions*:

(i) $\{\omega_{n}\}\subset [0, 1]$ *and* $\sum^{\infty }_{n=0}\omega _{n}=\infty $ *or*, *equivalently*, $\prod^{\infty }_{n=0}(1-\omega_{n})=0$;
(ii) $\limsup_{n\to \infty }\delta_{n}\leq 0$ *or* $\sum^{\infty }_{n=0} \omega_{n}\vert \delta_{n}\vert <\infty $;
(iii) $\gamma_{n}\geq 0$ ($n\geq 0$), $\sum^{\infty }_{n=0}\gamma_{n}< \infty $.

*Then* $\lim_{n\to \infty }s_{n}=0$.

### Lemma 2.3

(Demiclosedness principle [21])

*Let* *C* *be a nonempty closed convex subset of a real Hilbert space H*. *Let* $S: C\to C$ *be a nonexpansive mapping with* $\operatorname {Fix}(S)\neq \emptyset $. *Then the mapping* $I-S$ *is demiclosed*. *That is*, *if* $\{x_{n}\}$ *is a sequence in* *C* *such that* $x_{n}\rightharpoonup x^{*}$ *and* $(I-S)x_{n}\to y$, *then* $(I-S)x^{*}=y$. *Here* *I* *is the identity mapping of H*.

### Lemma 2.4

([22])

*Let* *H* *be a real Hilbert space*. *Let* $A: H\to H$ *be a strongly positive bounded linear operator with a constant* $\bar{\gamma }>1$. *Then*

$$ \bigl\langle (A-I)x-(A-I)y, x-y\bigr\rangle \geq (\bar{\gamma }-1) \Vert x-y \Vert ^{2}, \quad \forall x, y\in C. $$

*That is*, $A-I$ *is strongly monotone with a constant* $\bar{\gamma }-1$.

### Lemma 2.5

([22])

*Assume that* $A: H\to H$ *is a strongly positive bounded linear operator with a coefficient* $\bar{\gamma }>0$ *and* $0<\zeta \leq \Vert A\Vert ^{-1}$. *Then* $\Vert I-\zeta A\Vert \leq 1-\zeta \bar{ \gamma }$.

### Lemma 2.6

([23])

*Let* *C* *be a nonempty closed convex subset of a real Hilbert space H*. *Let* $G: C\to H$ *be a* *ρ*-*Lipschitzian and* *η*-*strongly monotone mapping with constants* $\rho , \eta >0$. *Let* $0<\mu <\frac{2\eta }{\rho^{2}}$ *and* $0< t<\sigma \leq 1$. *Then* $S:=\sigma I-t\mu G:C\to H$ *is a contractive mapping with constant* $\sigma - t\tau $, *where* $\tau =1- \sqrt{1-\mu (2\eta -\mu \rho^{2})}$.

### Lemma 2.7

([24])

*Let* *C* *be a nonempty closed convex subset of a real Hilbert space H*. *Let* $F: C\to H$ *be a continuous monotone mapping*. *Then*, *for* $r>0$ *and* $x\in H$, *there exists* $z\in C$ *such that*

$$ \langle y-z, Fz\rangle +\frac{1}{r}\langle y-z, z-x\rangle \geq 0, \quad \forall y\in C. $$

*For* $r>0$ *and* $x\in H$, *define* $F_{r}:H\to C$ *by*

$$ F_{r}x=\biggl\{ z\in C: \langle y-z, Fz\rangle +\frac{1}{r} \langle y-z, z-x \rangle \geq 0, \forall y\in C\biggr\} . $$

*Then the following hold*:

(i) $F_{r}$ *is single*-*valued*;
(ii) $F_{r}$ *is firmly nonexpansive*, *that is*,
  $$ \Vert F_{r}x-F_{r}y \Vert ^{2}\leq \langle x-y, F_{r}x-F_{r}y\rangle ,\quad \forall x, y\in H; $$
(iii) $\operatorname {Fix}(F_{r})=\operatorname {VI}(C, F)$;
(iv) $\operatorname {VI}(C, F)$ *is a closed convex subset of C*.

### Lemma 2.8

([24])

*Let* *C* *be a nonempty closed convex subset of a real Hilbert space H*. *Let* $T: C\to H$ *be a continuous pseudocontractive mapping*. *Then*, *for* $r>0$ *and* $x\in H$, *there exists* $z\in C$ *such that*

$$ \langle y-z, Tz\rangle -\frac{1}{r}\bigl\langle y-z,(1+r)z-x\bigr\rangle \leq 0, \quad \forall y\in C. $$

*For* $r>0$ *and* $x\in H$, *define* $T_{r}: H\to C$ *by*

$$ T_{r}x=\biggl\{ z\in C:\langle y-z,Tz\rangle -\frac{1}{r}\bigl\langle y-z,(1+r)z-x \bigr\rangle \leq 0, \forall y\in C\biggr\} . $$

*Then the following hold*:

(i) $T_{r}$ *is single*-*valued*;
(ii) $T_{r}$ *is firmly nonexpansive*, *that is*,
  $$ \Vert T_{r}x-T_{r}y \Vert ^{2}\leq \langle x-y, T_{r}x-T_{r}y\rangle ,\quad \forall x, y\in H; $$
(iii) $\operatorname {Fix}(T_{r})=\operatorname {Fix}(T)$;
(iv) $\operatorname {Fix}(T)$ *is a closed convex subset of C*.

## Main results

Throughout this section, we always assume the following:

- $B_{i}:C\to H$ is a $\mu_{i}$-inverse-strongly monotone mapping for each $i=1, 2,\ldots, N$;
- ${\varTheta }_{i}: C\times C\to {\mathbf{R}}$ is a bifunction satisfying conditions (A1)–(A4) for each $i=1, 2,\ldots, N$;
- $\varphi_{i}: C\to {\mathbf{R}}$ is a proper lower semicontinuous and convex function with restriction (B1) or (B2) for each $i=1, 2,\ldots, N$;
- $A: H\to H$ is a strongly positive linear bounded self-adjoint operator with a constant $\bar{\gamma }\in (1, 2)$;
- $V: C\to C$ is *l*-Lipschitzian with constant $l\in [0, \infty )$;
- $G: C\to C$ is a *ρ*-Lipschitzian and *η*-strongly monotone mapping with constants $\rho >0$ and $\eta >0$;
- constants *μ*, *l*, *τ*, and *γ* satisfy $0<\mu <\frac{2 \eta }{\rho^{2}}$ and $0\leq \gamma l<\tau $, where $\tau =1-\sqrt{1- \mu (2\eta -\mu \rho^{2})}$;
- $F_{1}, F_{2}: C\to H$ are continuous monotone mappings and $T:C\to C$ is a continuous pseudocontractive mapping such that ${\varOmega }:=\bigcap^{N}_{i=1}\operatorname {GMEP}({\varTheta }_{i}, \varphi _{i}, B_{i})\cap \operatorname {GSVI}(C, F_{1}, F_{2})\cap \operatorname {Fix}(T)\neq \emptyset $;
- $R_{t}=F_{1, \lambda_{t}}F_{2, \nu_{t}}:H\to C$, where $F_{1, \lambda _{t}}, F_{2, \nu_{t}}: H\to C$ are defined as follows:
  $$\begin{aligned}& F_{1, \lambda_{t}}x=\biggl\{ z\in C: \langle y-z, F_{1}z\rangle + \frac{1}{ \lambda_{t}}\langle y-z, z-x\rangle \geq 0, \forall y\in C\biggr\} , \\& F_{2, \nu_{t}}x=\biggl\{ z\in C: \langle y-z, F_{2}z\rangle + \frac{1}{\nu _{t}}\langle y-z,z-x\rangle \geq 0, \forall y\in C\biggr\} , \end{aligned}$$
  for $\lambda_{t}, \nu_{t}\in (0, \infty )$, $t\in (0, 1)$, $\lim_{t \to 0}\lambda_{t}=\lambda >0$, and $\lim_{t\to 0}\nu_{t}=\nu >0$;
- $R_{n}=F_{1, \lambda_{n}}F_{2, \nu_{n}}: H\to C$, where $F_{1, \lambda _{n}}, F_{2, \nu_{n}}: H\to C$ are defined as follows:
  $$\begin{aligned}& F_{1, \lambda_{n}}x=\biggl\{ z\in C: \langle y-z, F_{1}z\rangle + \frac{1}{ \lambda_{n}}\langle y-z, z-x\rangle \geq 0, \forall y\in C\biggr\} , \\& F_{2, \nu_{n}}x=\biggl\{ z\in C: \langle y-z, F_{2}z\rangle + \frac{1}{\nu _{n}}\langle y-z, z-x\rangle \geq 0, \forall y\in C\biggr\} , \end{aligned}$$
  for $\lambda_{n}, \nu_{n}\in (0, \infty )$, $\lim_{n\to \infty } \lambda_{n}=\lambda >0$, and $\lim_{n\to \infty }\nu_{n}=\nu >0$;
- $T_{r_{t}}: H\to C$ is a mapping defined by
  $$ T_{r_{t}}x=\biggl\{ z\in C: \langle y-z,Tz\rangle -\frac{1}{r_{t}}\bigl\langle y-z, (1+r_{t})z-x\bigr\rangle \geq 0, \forall y\in C\biggr\} $$
  for $r_{t}\in (0, \infty )$, $t\in (0, 1)$, and $\liminf_{t\to 0}r_{t}>0$;
- $T_{r_{n}}: H\to C$ is a mapping defined by
  $$ T_{r_{n}}x=\biggl\{ z\in C: \langle y-z,Tz\rangle -\frac{1}{r_{n}}\bigl\langle y-z, (1+r_{n})z-x\bigr\rangle \geq 0, \forall y\in C\biggr\} $$
  for $r_{n}\in (0, \infty )$, and $\liminf_{n\to \infty }r_{n}>0$;
- $T^{({\varTheta }_{i}, \varphi_{i})}_{r_{i, t}}:H\to C$ is a mapping defined by
  $$ T^{({\varTheta }_{i}, \varphi_{i})}_{r_{i, t}}x=\biggl\{ z\in C: {\varTheta }_{i}(z,y)+\varphi_{i}(y)-\varphi_{i}(z) + \frac{1}{r_{i, t}} \langle y-z,z-x\rangle \geq 0, \forall y\in C\biggr\} $$
  for $\{r_{i, t}\}_{t\in (0, 1)}\subset [c_{i}, d_{i}]\subset (0, 2\mu _{i})$ and $i\in \{1, 2,\ldots, N\}$;
- $T^{({\varTheta }_{i},\varphi_{i})}_{r_{i,n}}: H\to C$ is a mapping defined by
  $$ T^{({\varTheta }_{i},\varphi_{i})}_{r_{i, n}}x=\biggl\{ z\in C:{\varTheta }_{i}(z, y)+\varphi_{i}(y)-\varphi_{i}(z) + \frac{1}{r_{i, n}}\langle y-z, z-x\rangle \geq 0,\forall y\in C\biggr\} $$
  for $\{r_{i, n}\}^{\infty }_{n=1}\subset [c_{i}, d_{i}]\subset (0, 2 \mu_{i})$ and $i\in \{1, 2,\ldots, N\}$.

By Proposition 2.1 and Lemmas 2.7 and 2.8, we note that $T^{({\varTheta }_{i},\varphi_{i})}_{r_{i,t}}$, $T^{({\varTheta }_{i}, \varphi _{i})}_{r_{i,n}}$, $F_{1,\lambda_{t}}$, $F_{1,\lambda_{n}}$, $F_{2,\nu_{t}}$, $F _{2,\nu_{n}}$, $T_{r_{t}}$, and $T_{r_{n}}$ are nonexpansive, $\operatorname {GMEP}( {\varTheta }_{i},\varphi_{i},B_{i})=\operatorname {Fix}(T^{({\varTheta }_{i}, \varphi_{i})}_{r_{i,t}}(I-r_{i,t}B_{i}))=\operatorname {Fix}(T^{({\varTheta } _{i},\varphi_{i})}_{r_{i,n}}(I-r_{i,n}B_{i}))$, and $\operatorname {Fix}(T)= \operatorname {Fix}(T_{r_{t}})=\operatorname {Fix}(T_{r_{n}})$. So it is known that the composite mappings $R_{t}=F_{1,\lambda_{t}}F_{2,\nu_{t}}$ and $R_{n}=F_{1,\lambda_{n}}F_{2,\nu_{n}}$ are nonexpansive. Also, we note that $\operatorname {GSVI}(C,F_{1},F_{2})=\operatorname {Fix}(R_{t})=\operatorname {Fix}(R_{n})$ by Lemma 1.1.

In this section, for $t\in (0, 1)$, $n\geq 1$ and $i\in \{1, 2,\ldots, N \}$, we put

$$\begin{aligned}& {{\varDelta}}^{i}_{t}=T^{({\varTheta }_{i},\varphi_{i})}_{r_{i,t}}(I-r _{i,t}B_{i})T^{({\varTheta }_{i-1}, \varphi_{i-1})}_{r_{i-1,t}}(I-r _{i-1, t}B_{i-1})\cdots T^{({\varTheta }_{1},\varphi_{1})}_{r_{1,t}}(I-r _{1, t}B_{1}), \\& {{\varDelta}}^{i}_{n}=T^{({\varTheta }_{i},\varphi_{i})}_{r_{i, n}}(I-r _{i, n}B_{i})T^{({\varTheta }_{i-1}, \varphi_{i-1})}_{r_{i-1,n}}(I-r _{i-1,n}B_{i-1})\cdots T^{({\varTheta }_{1}, \varphi_{1})}_{r_{1, n}}(I-r _{1,n}B_{1}), \end{aligned}$$

and ${\varDelta}^{0}_{t}={\varDelta}^{0}_{n}=I$.

We now introduce the first general iterative scheme that generates a net $\{x_{t}\}$ in an implicit way:

3.1 $$ x_{t}=P_{C}\bigl[(I-\theta_{t}A)T_{r_{t}}{ {\varDelta}}^{N}_{t}R_{t}x_{t}+ \theta_{t}\bigl(t\gamma Vx_{t}+(I-t\mu G)T_{r_{t}}{ {\varDelta}}^{N}_{t}R _{t}x_{t} \bigr)\bigr], $$

where $t\in (0,\min \{1, \frac{2-\bar{\gamma }}{\tau -\gamma l}\})$ and $\theta_{t}\in (0,\min \{\frac{1}{2}, \Vert A\Vert ^{-1}\})$.

We prove the strong convergence of $\{x_{t}\}$ as $t\to 0$ to a point $\widetilde{x}\in {{\varOmega }}$, which is a unique solution to the VI

3.2 $$ \bigl\langle (A-I)\widetilde{x}, p-\widetilde{x}\bigr\rangle \geq 0, \quad \forall p\in {{\varOmega }}. $$

In the meantime, we also propose the second general iterative scheme that generates a sequence $\{x_{n}\}$ in an explicit way:

3.3 $$ \textstyle\begin{cases} w_{n}=\alpha_{n}\gamma Vx_{n}+(I-\alpha_{n}\mu G)T_{r_{n}}{{\varDelta}}^{N}_{n}R_{n}x_{n}, \\ x_{n+1}=P_{C}[(I-\beta_{n}A)T_{r_{n}}{{\varDelta}}^{N}_{n}R_{n}x_{n}+ \beta_{n}w_{n}],\quad \forall n\geq 1, \end{cases} $$

where $\{\alpha_{n}\}, \{\beta_{n}\}\subset [0, 1]$ and $x_{0}\in C$ is an arbitrary initial guess, and establish the strong convergence of $\{x_{n}\}$ as $n\to \infty $ to the same point $\widetilde{x}\in {{\varOmega }}$, which is the unique solution to VI (3.2).

Next, for $t\in (0,\min \{1, \frac{2-\bar{\gamma }}{\tau -\gamma l}\})$ and $\theta_{t}\in (0, \min \{\frac{1}{2},\Vert A\Vert ^{-1}\})$, consider a mapping $Q_{t}: C\to C$ defined by

$$ Q_{t}x=P_{C}\bigl[(I-\theta_{t}A)T_{r_{t}}{ {\varDelta}}^{N}_{t}R_{t}x+ \theta_{t}\bigl(t\gamma Vx+(I-t\mu G)T_{r_{t}}{{\varDelta}}^{N}_{t}R_{t}x\bigr)\bigr], \quad \forall x\in C. $$

It is easy to see that $Q_{t}$ is a contractive mapping with constant $1-\theta_{t}(\bar{\gamma }-1+t(\tau -\gamma l))$. Indeed, by Propositions 2.1 and 2.2 and Lemmas 2.5 and 2.6, we have

$$\begin{aligned} \Vert Q_{t}x-Q_{t}y \Vert &\leq \bigl\Vert (I- \theta_{t}A)T_{r_{t}}{{\varDelta}}^{N} _{t}R_{t}x+\theta_{t}\bigl(t\gamma Vx+(I-t\mu G)T_{r_{t}}{{\varDelta}}^{N} _{t}R_{t}x \bigr) \\ &\quad{} -(I-\theta_{t}A)T_{r_{t}}{{\varDelta}}^{N}_{t}R_{t}y-\theta_{t}\bigl(t \gamma Vy+(I-t\mu G)T_{r_{t}}{{\varDelta}}^{N}_{t}R_{t}y \bigr) \bigr\Vert \\ &\leq \bigl\Vert (I-\theta_{t}A)T_{r_{t}}{{\varDelta}}^{N}_{t}R_{t}x-(I-\theta _{t}A)T_{r_{t}}{ {\varDelta}}^{N}_{t}R_{t}y \bigr\Vert \\ & \quad{}+\theta_{t} \bigl\Vert \bigl(t\gamma Vx+(I-t\mu G)T_{r_{t}}{{\varDelta}}^{N}_{t}R _{t}x\bigr)-\bigl(t\gamma Vy+(I-t\mu G)T_{r_{t}}{{\varDelta}}^{N}_{t}R_{t}y\bigr) \bigr\Vert \\ &\leq (1-\theta_{t}\bar{\gamma }) \bigl\Vert T_{r_{t}}{ {\varDelta}}^{N}_{t}R _{t}x-T_{r_{t}}{ {\varDelta}}^{N}_{t}R_{t}y \bigr\Vert + \theta_{t}\bigl[t\gamma \Vert Vx-Vy \Vert \\ & \quad{}+ \bigl\Vert (I-t\mu G)T_{r_{t}}{{\varDelta}}^{N}_{t}R_{t}x-(I-t\mu G)T_{r_{t}} { {\varDelta}}^{N}_{t}R_{t}y \bigr\Vert \bigr] \\ &\leq (1-\theta_{t}\bar{\gamma }) \Vert x-y \Vert + \theta_{t}\bigl[t\gamma l \Vert x-y \Vert +(1-t \tau ) \Vert x-y \Vert \bigr] \\ &=\bigl[1-\theta_{t}\bigl(\bar{\gamma }-1+t(\tau -\gamma l)\bigr) \bigr] \Vert x-y \Vert . \end{aligned}$$

Since $\bar{\gamma }\in (1, 2)$, $\tau -\gamma l>0$ and $0< t<\min \{1, \frac{2-\bar{ \gamma }}{\tau -\gamma l}\}\leq \frac{2-\bar{\gamma }}{\tau -\gamma l}$, it follows that $0<\bar{ \gamma }-1+t(\tau -\gamma l)<1$, which together with $0<\theta_{t}< \min \{\frac{1}{2},\Vert A\Vert ^{-1}\}<1$ yields $0<1-\theta_{t}(\bar{\gamma }-1+t(\tau -\gamma l))<1$. Hence $Q_{t}$ is a contractive mapping. By the Banach contraction principle, $Q_{t}$ has a unique fixed point, denoted by $x_{t}$, which uniquely solves the fixed point equation (3.1).

We summarize the basic properties of $\{x_{t}\}$.

### Theorem 3.1

*Let* $\{x_{t}\}$ *be defined via* (3.1). *Then*

(i) $\{x_{t}\}$ *is bounded for* $t\in (0, \min \{1, \frac{2-\bar{ \gamma }}{\tau -\gamma l}\})$;
(ii) $\lim_{t\to 0}\Vert x_{t}-R_{t}x_{t}\Vert =0$, $\lim_{t\to 0}\Vert x_{t}-{\varDelta}^{N}_{t}x_{t}\Vert =0$, *and* $\lim_{t\to 0}\Vert x_{t}-T_{r_{t}}x_{t} \Vert =0$ *provided* $\lim_{t\to 0}\theta_{t}=0$;
(iii) $x_{t}: (0, \min \{1,\frac{2-\bar{\gamma }}{\tau -\gamma l}\}) \to H$ *is locally Lipschitzian provided* $\theta_{t}: (0,\min \{1,\frac{2-\bar{ \gamma }}{\tau -\gamma l}\})\to (0,\min \{\frac{1}{2},\Vert A\Vert ^{-1}\})$ *is locally Lipschitzian*, $r_{t}, \lambda_{t},\nu_{t}: (0,\min \{1, \frac{2-\bar{ \gamma }}{\tau -\gamma l}\})\to (0, \infty )$ *are locally Lipschitzian*, *and* $r_{i,t}: (0, \min \{1, \frac{2-\bar{\gamma }}{\tau -\gamma l}\}) \to [c_{i}, d_{i}]$ *is locally Lipschitzian for each* $i=1, 2,\ldots, N$;
(iv) $x_{t}$ *defines a continuous path from* $(0,\min \{1,\frac{2-\bar{ \gamma }}{\tau -\gamma l}\})$ *into* *H* *provided* $\theta_{t}: (0, \min \{1, \frac{2-\bar{\gamma }}{\tau -\gamma l}\})\to (0,\min \{ \frac{1}{2}, \Vert A\Vert ^{-1}\})$ *is continuous*, $r_{t},\lambda_{t},\nu_{t}: (0, \min \{1, \frac{2-\bar{\gamma }}{\tau -\gamma l}\})\to (0, \infty )$ *are continuous*, *and* $r_{i, t}: (0,\min \{1,\frac{2-\bar{\gamma }}{ \tau -\gamma l}\})\to [c_{i}, d_{i}]$ *is continuous for each* $i=1, 2,\ldots, N$.

### Proof

Let $z_{t}=R_{t}x_{t}$, $u_{t}={\varDelta}^{N}_{t}z _{t}$, and $v_{t}=T_{r_{t}}u_{t}$. Take $p\in \varOmega $. Then $p=T_{r_{t}}p$ by Lemma 2.8(iii), $p={\varDelta}^{i}_{t}p$ ($=T^{( {\varTheta }_{i},\varphi_{i})}_{r_{i,t}}(I-r_{i, t}B_{i})p$) by Proposition 2.1(iii), and $p=R_{t}p$ by Lemma 1.1.

(i) Utilizing Proposition 2.1(ii) and Proposition 2.2, we have

3.4 $$\begin{aligned} \Vert u_{t}-p \Vert &= \bigl\Vert T^{({\varTheta }_{N},\varphi_{N})}_{r_{N,t}}(I-r_{N,t}B _{N}){\varDelta}^{N-1}_{t}z_{t} -T^{({\varTheta }_{N},\varphi_{N})} _{r_{N,t}}(I-r_{N,t}B_{N}){\varDelta}^{N-1}_{t}p \bigr\Vert \\ &\leq \bigl\Vert (I-r_{N, t}B_{N}){\varDelta}^{N-1}_{t}z_{t}-(I-r_{N,t}B_{N}) {\varDelta}^{N-1}_{t}p \bigr\Vert \\ &\leq \bigl\Vert {{\varDelta}}^{N-1}_{t}z_{t}- {\varDelta}^{N-1}_{t}p \bigr\Vert \\ &\leq \cdots \\ &\leq \bigl\Vert {{\varDelta}}^{0}_{t}z_{t}- {\varDelta}^{0}_{t}p \bigr\Vert = \Vert z_{t}-p \Vert . \end{aligned}$$

Moreover, it is easy from the nonexpansivity of $R_{t}$ to see that

$$ \Vert z_{t}-p \Vert = \Vert R_{t}x_{t}-R_{t}p \Vert \leq \Vert x_{t}-p \Vert , $$

which together with the nonexpansivity of $T_{r_{t}}$ and (3.4) implies that

3.5 $$ \Vert v_{t}-p \Vert = \Vert T_{r_{t}}u_{t}-T_{r_{t}}p \Vert \leq \Vert u_{t}-p \Vert \leq \Vert z_{t}-p \Vert \leq \Vert x_{t}-p \Vert . $$

By (3.5), we have

$$\begin{aligned} \Vert x_{t}-p \Vert &\leq \bigl\Vert (I-\theta_{t}A)v_{t}+ \theta_{t}\bigl(t\gamma Vx_{t}+(I-t \mu G)v_{t} \bigr)-p \bigr\Vert \\ &= \bigl\Vert (I-\theta_{t}A)v_{t}-(I- \theta_{t}A)p+\theta_{t}\bigl(t\gamma Vx_{t}+(I-t \mu G)v_{t}-p\bigr)+\theta_{t}(I-A)p \bigr\Vert \\ &\leq \bigl\Vert (I-\theta_{t}A)v_{t}-(I- \theta_{t}A)p \bigr\Vert +\theta_{t} \bigl\Vert t\gamma Vx _{t}+(I-t\mu G)v_{t}-p \bigr\Vert + \theta_{t} \bigl\Vert (I-A)p \bigr\Vert \\ &= \bigl\Vert (I-\theta_{t}A)v_{t}-(I- \theta_{t}A)p \bigr\Vert \\ & \quad{}+\theta_{t} \bigl\Vert (I-t\mu G)v_{t}-(I-t\mu G)p+t(\gamma Vx_{t}-\mu Gp) \bigr\Vert + \theta_{t} \bigl\Vert (I-A)p \bigr\Vert \\ &\leq (1-\theta_{t}\bar{\gamma }) \Vert v_{t}-p \Vert +\theta_{t}\bigl[ \bigl\Vert (I-t\mu G)v _{t}-(I-t\mu G)p \bigr\Vert \\ & \quad{}+t\bigl(\gamma \Vert Vx_{t}-Vp \Vert + \Vert \gamma Vp-\mu Gp \Vert \bigr)\bigr]+\theta_{t} \bigl\Vert (I-A)p \bigr\Vert \\ &\leq (1-\theta_{t}\bar{\gamma }) \Vert x_{t}-p \Vert +\theta_{t}\bigl[(1-t\tau ) \Vert x _{t}-p \Vert +t \bigl(\gamma l \Vert x_{t}-p \Vert + \bigl\Vert (\gamma V-\mu G)p \bigr\Vert \bigr)\bigr] \\ & \quad{}+\theta_{t} \Vert I-A \Vert \Vert p \Vert \\ &=\bigl[1-\theta_{t}\bigl(\bar{\gamma }-1+t(\tau -\gamma l)\bigr) \bigr] \Vert x_{t}-p \Vert +\theta _{t}\bigl[ \Vert I-A \Vert \Vert p \Vert +t \bigl\Vert (\gamma V-\mu G)p \bigr\Vert \bigr]. \end{aligned}$$

So, it follows that

$$ \Vert x_{t}-p \Vert \leq \frac{ \Vert I-A \Vert \Vert p \Vert +t \Vert (\gamma V-\mu G)p \Vert }{\bar{ \gamma }-1+t(\tau -\gamma l)} \leq \frac{ \Vert I-A \Vert \Vert p \Vert + \Vert (\gamma V- \mu G)p \Vert }{\bar{\gamma }-1}. $$

Hence $\{x_{t}\}$ is bounded and so are $\{Vx_{t}\}$, $\{u_{t}\}$, $\{v _{t}\}$, $\{z_{t}\}$, and $\{Gv_{t}\}$.

(ii) By the definition of $\{x_{t}\}$, we have

$$\begin{aligned} \Vert x_{t}-v_{t} \Vert &= \bigl\Vert P_{C}\bigl[(I-\theta_{t}A)v_{t}+ \theta_{t}\bigl(t\gamma Vx _{t}+(I-t\mu G)v_{t} \bigr)\bigr]-v_{t} \bigr\Vert \\ &\leq \bigl\Vert (I-\theta_{t}A)v_{t}+ \theta_{t}\bigl(t\gamma Vx_{t}+(I-t\mu G)v _{t} \bigr)-v_{t} \bigr\Vert \\ &= \bigl\Vert \theta_{t}\bigl[(I-A)v_{t}+t(\gamma Vx_{t}-\mu Gv_{t})\bigr] \bigr\Vert \\ &=\theta_{t} \bigl\Vert (I-A)v_{t}+t(\gamma Vx_{t}-\mu Gv_{t}) \bigr\Vert \\ &\leq \theta_{t} \Vert I-A \Vert \Vert v_{t} \Vert +t \Vert \gamma Vx_{t}-\mu Gv_{t} \Vert \to 0 \quad \text{as } t\to 0, \end{aligned}$$

using the boundedness of $\{Vx_{t}\}$, $\{v_{t}\}$, and $\{Gv_{t}\}$ in the proof of assertion (i). That is,

3.6 $$ \lim_{t\to 0} \Vert x_{t}-v_{t} \Vert =0. $$

In view of (3.5) and Lemma 2.7(ii), we get

$$\begin{aligned} \Vert v_{t}-p \Vert ^{2}&\leq \Vert z_{t}-p \Vert ^{2}= \Vert R_{t}x_{t}-R_{t}p \Vert ^{2} \\ &= \Vert F_{1,\lambda_{t}}F_{2, \nu_{t}}x_{t}-F_{1,\lambda_{t}}F_{2, \nu _{t}}p \Vert ^{2} \\ &\leq \langle F_{2,\nu_{t}}x_{t}-F_{2, \nu_{t}}p,F_{1,\lambda_{t}}F _{2, \nu_{t}}x_{t}-F_{1,\lambda_{t}}F_{2, \nu_{t}}p\rangle \\ &=\langle F_{2, \nu_{t}}x_{t}-F_{2,\nu_{t}}p,z_{t}-p \rangle \\ &\leq \frac{1}{2}\bigl[ \Vert F_{2, \nu_{t}}x_{t}-F_{2,\nu_{t}}p \Vert ^{2}+ \Vert z_{t}-p \Vert ^{2}- \bigl\Vert (F_{2, \nu_{t}}x_{t}-F_{2, \nu_{t}}p)-(z_{t}-p) \bigr\Vert ^{2}\bigr] \\ &\leq \frac{1}{2}\bigl[ \Vert x_{t}-p \Vert ^{2}+ \Vert x_{t}-p \Vert ^{2}- \bigl\Vert (F_{2, \nu_{t}}x _{t}-F_{2,\nu_{t}}p)-(z_{t}-p) \bigr\Vert ^{2}\bigr] \\ &= \Vert x_{t}-p \Vert ^{2}-\frac{1}{2} \bigl\Vert (F_{2, \nu_{t}}x_{t}-F_{2, \nu_{t}}p)-(z _{t}-p) \bigr\Vert ^{2}, \end{aligned}$$

which immediately yields

$$ \frac{1}{2} \bigl\Vert (F_{2, \nu_{t}}x_{t}-F_{2, \nu_{t}}p)-(z_{t}-p) \bigr\Vert ^{2} \leq \Vert x_{t}-p \Vert ^{2}- \Vert v_{t}-p \Vert ^{2} \leq \bigl( \Vert x_{t}-p \Vert + \Vert v_{t}-p \Vert \bigr) \Vert x _{t}-v_{t} \Vert . $$

From (3.6) and the boundedness of $\{x_{t}\}$ and $\{v_{t}\}$, we have

3.7 $$ \lim_{t\to 0} \bigl\Vert (F_{2,\nu_{t}}x_{t}-F_{2,\nu_{t}}p)-(z_{t}-p) \bigr\Vert =0. $$

Again from (3.5) and Lemma 2.7(ii), we obtain

$$\begin{aligned} \Vert v_{t}-p \Vert ^{2}&\leq \Vert z_{t}-p \Vert ^{2}= \Vert R_{t}x_{t}-R_{t}p \Vert ^{2} \\ &\leq \Vert F_{2, \nu_{t}}x_{t}-F_{2,\nu_{t}}p \Vert ^{2} \\ &\leq \langle x_{t}-p, F_{2,\nu_{t}}x_{t}-F_{2,\nu_{t}}p \rangle \\ &\leq \frac{1}{2}\bigl[ \Vert x_{t}-p \Vert ^{2}+ \Vert F_{2, \nu_{t}}x_{t}-F_{2,\nu_{t}}p \Vert ^{2}- \bigl\Vert (x_{t}-p)-(F_{2, \nu_{t}}x_{t}-F_{2, \nu_{t}}p) \bigr\Vert ^{2}\bigr] \\ &\leq \frac{1}{2}\bigl[ \Vert x_{t}-p \Vert ^{2}+ \Vert x_{t}-p \Vert ^{2}- \bigl\Vert (x_{t}-p)-(F_{2, \nu_{t}}x_{t}-F_{2,\nu_{t}}p) \bigr\Vert ^{2}\bigr] \\ &= \Vert x_{t}-p \Vert ^{2}-\frac{1}{2} \bigl\Vert (x_{t}-p)-(F_{2,\nu_{t}}x_{t}-F_{2,\nu _{t}}p) \bigr\Vert ^{2}, \end{aligned}$$

which hence leads to

$$ \frac{1}{2} \bigl\Vert (x_{t}-p)-(F_{2,\nu_{t}}x_{t}-F_{2,\nu_{t}}p) \bigr\Vert ^{2} \leq \Vert x_{t}-p \Vert ^{2}- \Vert v_{t}-p \Vert ^{2} \leq \bigl( \Vert x_{t}-p \Vert + \Vert v_{t}-p \Vert \bigr) \Vert x_{t}-v_{t} \Vert . $$

Again from (3.6) and the boundedness of $\{x_{t}\}$ and $\{v_{t}\}$, we have

3.8 $$ \lim_{t\to 0} \bigl\Vert (x_{t}-p)-(F_{2,\nu_{t}}x_{t}-F_{2,\nu_{t}}p) \bigr\Vert =0. $$

So it follows from (3.7) and (3.8) that

$$ \Vert x_{t}-z_{t} \Vert \leq \bigl\Vert (x_{t}-p)-(F_{2,\nu_{t}}x_{t}-F_{2,\nu_{t}}p) \bigr\Vert + \bigl\Vert (F_{2,\nu_{t}}x_{t}-F_{2,\nu_{t}}p)-(z_{t}-p) \bigr\Vert \to 0\quad \text{as } t \to 0. $$

That is,

3.9 $$ \lim_{t\to 0} \Vert x_{t}-z_{t} \Vert =0. $$

Furthermore, from (3.5) and Proposition 2.1(ii) and Proposition 2.2, it follows that

$$\begin{aligned} \Vert v_{t}-p \Vert ^{2}&\leq \Vert u_{t}-p \Vert ^{2}= \bigl\Vert {{\varDelta}}^{N}_{t}z_{t}-p \bigr\Vert ^{2} \\ &\leq \bigl\Vert {{\varDelta}}^{i}_{t}z_{t}-p \bigr\Vert ^{2} \\ &= \bigl\Vert T^{({\varTheta }_{i}, \varphi_{i})}_{r_{i,t}}(I-r_{i,t}B_{i}) {\varDelta}^{i-1}_{t}z_{t} -T^{({\varTheta }_{i}, \varphi_{i})} _{r_{i,t}}(I-r_{i, t}B_{i})p \bigr\Vert ^{2} \\ &\leq \bigl\Vert (I-r_{i,t}B_{i}){\varDelta}^{i-1}_{t}z_{t}-(I-r_{i, t}B_{i})p \bigr\Vert ^{2} \\ &\leq \bigl\Vert {{\varDelta}}^{i-1}_{t}z_{t}-p \bigr\Vert ^{2}+r_{i,t}(r_{i,t}-2 \mu_{i}) \bigl\Vert B_{i}{{\varDelta}}^{i-1}_{t}z_{t}-B_{i}p \bigr\Vert ^{2} \\ &\leq \Vert z_{t}-p \Vert ^{2}+r_{i, t}(r_{i,t}-2 \mu_{i}) \bigl\Vert B_{i}{{\varDelta}} ^{i-1}_{t}z_{t}-B_{i}p \bigr\Vert ^{2} \\ &\leq \Vert x_{t}-p \Vert ^{2}+r_{i, t}(r_{i,t}-2 \mu_{i}) \bigl\Vert B_{i}{{\varDelta}} ^{i-1}_{t}z_{t}-B_{i}p \bigr\Vert ^{2}, \end{aligned}$$

which together with $\{r_{i, t}\}_{t\in (0,1)}\subset [c_{i},d_{i}] \subset (0,2\mu_{i})$ for $i\in \{1,2,\ldots,N\}$ implies that

$$\begin{aligned} c_{i}(2\mu_{i}-d_{i}) \bigl\Vert B_{i}{{\varDelta}}^{i-1}_{t}z_{t}-B_{i}p \bigr\Vert ^{2} &\leq r_{i,t}(2\mu_{i}-r_{i, t}) \bigl\Vert B_{i}{{\varDelta}}^{i-1}_{t}z_{t}-B _{i}p \bigr\Vert ^{2} \\ &\leq \Vert x_{t}-p \Vert ^{2}- \Vert v_{t}-p \Vert ^{2}\leq \bigl( \Vert x_{t}-p \Vert + \Vert v_{t}-p \Vert \bigr) \Vert x _{t}-v_{t} \Vert . \end{aligned}$$

From (3.6) and the boundedness of $\{x_{t}\}$ and $\{v_{t}\}$, we have

3.10 $$ \lim_{t\to 0} \bigl\Vert B_{i}{{\varDelta}}^{i-1}_{t}z_{t}-B_{i}p \bigr\Vert =0. $$

Also, by Proposition 2.1(ii), we obtain that, for each $i=1, 2,\ldots, N$,

$$\begin{aligned}& \bigl\Vert {{\varDelta}}^{i}_{t}z_{t}-p \bigr\Vert ^{2} \\ & \quad = \bigl\Vert T^{({\varTheta }_{i},\varphi_{i})}_{r_{i,t}}(I-r_{i,t}B_{i}) {\varDelta}^{i-1}_{t}z_{t} -T^{({\varTheta }_{i},\varphi_{i})}_{r _{i,t}}(I-r_{i,t}B_{i})p \bigr\Vert ^{2} \\ & \quad \leq \bigl\langle (I-r_{i,t}B_{i}){\varDelta}^{i-1}_{t}z_{t}-(I-r_{i,t}B _{i})p,{\varDelta}^{i}_{t}z_{t}-p \bigr\rangle \\ & \quad =\frac{1}{2}\bigl[ \bigl\Vert (I-r_{i,t}B_{i}) {\varDelta}^{i-1}_{t}z_{t}-(I-r_{i,t}B _{i})p \bigr\Vert ^{2}+ \bigl\Vert {{\varDelta}}^{i}_{t}z_{t}-p \bigr\Vert ^{2} \\ & \quad \quad{}- \bigl\Vert (I-r_{i,t}B_{i}){\varDelta}^{i-1}_{t}z_{t}-(I-r_{i,t}B_{i})p- \bigl( {\varDelta}^{i}_{t}z_{t}-p\bigr) \bigr\Vert ^{2}\bigr] \\ & \quad \leq \frac{1}{2}\bigl[ \bigl\Vert {{\varDelta}}^{i-1}_{t}z_{t}-p \bigr\Vert ^{2}+ \bigl\Vert {{\varDelta}}^{i}_{t}z_{t}-p \bigr\Vert ^{2} - \bigl\Vert {{\varDelta}}^{i-1}_{t}z_{t}- {\varDelta}^{i}_{t}z_{t}-r_{i,t} \bigl(B_{i}{{\varDelta}}^{i-1}_{t}z_{t}-B_{i}p \bigr) \bigr\Vert ^{2}\bigr] \\ & \quad \leq \frac{1}{2}\bigl[ \Vert x_{t}-p \Vert ^{2}+ \bigl\Vert {{\varDelta}}^{i}_{t}z_{t}-p \bigr\Vert ^{2} - \bigl\Vert {{\varDelta}}^{i-1}_{t}z_{t}- {\varDelta}^{i}_{t}z_{t}-r_{i,t} \bigl(B _{i}{{\varDelta}}^{i-1}_{t}z_{t}-B_{i}p \bigr) \bigr\Vert ^{2}\bigr], \end{aligned}$$

which immediately implies that

$$ \bigl\Vert {{\varDelta}}^{i}_{t}z_{t}-p \bigr\Vert ^{2}\leq \Vert x_{t}-p \Vert ^{2} - \bigl\Vert {{\varDelta}}^{i-1}_{t}z_{t}- {\varDelta}^{i}_{t}z_{t}-r_{i,t} \bigl(B_{i}{{\varDelta}}^{i-1}_{t}z_{t}-B_{i}p \bigr) \bigr\Vert ^{2}. $$

This together with (3.5) leads to

$$\begin{aligned} \Vert v_{t}-p \Vert ^{2}&\leq \Vert u_{t}-p \Vert ^{2}= \bigl\Vert {{\varDelta}}^{N}_{t}z_{t}-p \bigr\Vert ^{2}\leq \bigl\Vert {{\varDelta}}^{i}_{t}z_{t}-p \bigr\Vert ^{2} \\ &\leq \Vert x_{t}-p \Vert ^{2}- \bigl\Vert {{\varDelta}}^{i-1}_{t}z_{t}-{\varDelta} ^{i}_{t}z_{t}-r_{i, t} \bigl(B_{i}{{\varDelta}}^{i-1}_{t}z_{t}-B_{i}p \bigr) \bigr\Vert ^{2}, \end{aligned}$$

which hence implies

$$ \begin{aligned} \bigl\Vert {{\varDelta}}^{i-1}_{t}z_{t}- {\varDelta}^{i}_{t}z_{t}-r_{i,t} \bigl(B _{i}{{\varDelta}}^{i-1}_{t}z_{t}-B_{i}p \bigr) \bigr\Vert ^{2} &\leq \Vert x_{t}-p \Vert ^{2}- \Vert v_{t}-p \Vert ^{2} \\&\leq \bigl( \Vert x_{t}-p \Vert + \Vert v_{t}-p \Vert \bigr) \Vert x_{t}-v_{t} \Vert .\end{aligned} $$

From (3.6) and the boundedness of $\{x_{t}\}$ and $\{v_{t}\}$, we have

$$ \lim_{t\to 0} \bigl\Vert {{\varDelta}}^{i-1}_{t}z_{t}- {\varDelta}^{i}_{t}z _{t}-r_{i, t} \bigl(B_{i}{{\varDelta}}^{i-1}_{t}z_{t}-B_{i}p \bigr) \bigr\Vert =0, $$

which together with (3.10) implies that, for each $i=1, 2,\ldots, N$,

3.11 $$ \lim_{t\to 0} \bigl\Vert {{\varDelta}}^{i-1}_{t}z_{t}- {\varDelta}^{i}_{t}z _{t} \bigr\Vert =0. $$

Note that

$$ \Vert z_{t}-u_{t} \Vert \leq \sum ^{N}_{i=1} \bigl\Vert {{\varDelta}}^{i-1}_{t}z_{t}- {\varDelta}^{i}_{t}z_{t} \bigr\Vert . $$

From (3.11), it is easy to see that

3.12 $$ \lim_{t\to 0} \Vert z_{t}-u_{t} \Vert =0. $$

Also, observe that

$$\begin{aligned} \bigl\Vert x_{t}-{\varDelta}^{N}_{t}x_{t} \bigr\Vert &\leq \Vert x_{t}-z_{t} \Vert + \bigl\Vert z_{t}- {\varDelta}^{N}_{t}z_{t} \bigr\Vert + \bigl\Vert {{\varDelta}}^{N}_{t}z_{t}- {\varDelta}^{N}_{t}x_{t} \bigr\Vert \\ &\leq \Vert x_{t}-z_{t} \Vert + \bigl\Vert z_{t}-{\varDelta}^{N}_{t}z_{t} \bigr\Vert + \Vert z_{t}-x _{t} \Vert \\ &=2 \Vert x_{t}-z_{t} \Vert + \Vert z_{t}-u_{t} \Vert . \end{aligned}$$

From (3.9) and (3.12), it is easy to see that

3.13 $$ \lim_{t\to 0} \bigl\Vert x_{t}-{\varDelta}^{N}_{t}x_{t} \bigr\Vert =0. $$

In the meantime, again from (3.5) and Lemma 2.7(ii), we obtain

$$\begin{aligned} \Vert v_{t}-p \Vert ^{2}&= \Vert T_{r_{t}}u_{t}-T_{r_{t}}p \Vert ^{2} \\ &\leq \langle u_{t}-p, T_{r_{t}}u_{t}-T_{r_{t}}p \rangle =\langle u _{t}-p, v_{t}-p\rangle \\ &=\frac{1}{2}\bigl[ \Vert u_{t}-p \Vert ^{2}+ \Vert v_{t}-p \Vert ^{2}- \bigl\Vert u_{t}-p-(v_{t}-p) \bigr\Vert ^{2}\bigr] \\ &\leq \frac{1}{2}\bigl[ \Vert x_{t}-p \Vert ^{2}+ \Vert x_{t}-p \Vert ^{2}- \Vert u_{t}-v_{t} \Vert ^{2}\bigr] \\ &= \Vert x_{t}-p \Vert ^{2}-\frac{1}{2} \Vert u_{t}-v_{t} \Vert ^{2}, \end{aligned}$$

which immediately yields

$$ \frac{1}{2} \Vert u_{t}-v_{t} \Vert ^{2}\leq \Vert x_{t}-p \Vert ^{2}- \Vert v_{t}-p \Vert ^{2} \leq \bigl( \Vert x_{t}-p \Vert + \Vert v_{t}-p \Vert \bigr) \Vert x_{t}-v_{t} \Vert . $$

From (3.6) and the boundedness of $\{x_{t}\}$ and $\{v_{t}\}$, we have

3.14 $$ \lim_{t\to 0} \Vert u_{t}-v_{t} \Vert =0. $$

Taking into account that

$$\begin{aligned} \Vert x_{t}-T_{r_{t}}x_{t} \Vert &\leq \Vert x_{t}-u_{t} \Vert + \Vert u_{t}-T_{r_{t}}u_{t} \Vert + \Vert T_{r_{t}}u_{t}-T_{r_{t}}x_{t} \Vert \\ &\leq \Vert x_{t}-u_{t} \Vert + \Vert u_{t}-T_{r_{t}}u_{t} \Vert + \Vert u_{t}-x_{t} \Vert \\ &=2 \Vert x_{t}-u_{t} \Vert + \Vert u_{t}-v_{t} \Vert \\ &\leq 2\bigl( \Vert x_{t}-z_{t} \Vert + \Vert z_{t}-u_{t} \Vert \bigr)+ \Vert u_{t}-v_{t} \Vert , \end{aligned}$$

we deduce from (3.9), (3.12), and (3.14) that

3.15 $$ \lim_{t\to 0} \Vert x_{t}-T_{r_{t}}x_{t} \Vert =0. $$

(iii) Let $t,t_{0}\in (0,\min \{1,\frac{2-\bar{\gamma }}{\tau -\gamma l}\})$. Since $v_{t}=T_{r_{t}}u_{t}$ and $v_{t_{0}}=T_{r_{t_{0}}} u _{t_{0}}$, we get

3.16 $$ \bigl\langle y-v_{t}, (I-T)v_{t}\bigr\rangle + \frac{1}{r_{t}}\langle y-v_{t}, v _{t}-u_{t} \rangle \geq 0,\quad \forall y\in C, $$

and

3.17 $$ \bigl\langle y-v_{t_{0}},(I-T)v_{t_{0}}\bigr\rangle + \frac{1}{r_{t_{0}}}\langle y-v_{t_{0}},v_{t_{0}}-u_{t_{0}} \rangle \geq 0,\quad \forall y\in C. $$

Putting $y=v_{t_{0}}$ in (3.16) and $y=v_{t}$ in (3.17), we obtain

3.18 $$ \bigl\langle v_{t_{0}}-v_{t}, (I-T)v_{t}\bigr\rangle +\frac{1}{r_{t}}\langle v _{t_{0}}-v_{t}, v_{t}-u_{t}\rangle \geq 0 $$

and

3.19 $$ \bigl\langle v_{t}-v_{t_{0}}, (I-T)v_{t_{0}}\bigr\rangle +\frac{1}{r_{t_{0}}} \langle v_{t}-v_{t_{0}}, v_{t_{0}}-u_{t_{0}}\rangle \geq 0. $$

Adding up (3.18) and (3.19), we have

$$ -\bigl\langle v_{t}-v_{t_{0}}, (I-T)v_{t}-(I-T)v_{t_{0}} \bigr\rangle +\biggl\langle v _{t_{0}}-v_{t},\frac{v_{t}-u_{t}}{r_{t}}- \frac{v_{t_{0}}-u_{t_{0}}}{r _{t_{0}}}\biggr\rangle \geq 0. $$

Since *T* is pseudocontractive, we know that $I-T$ is a monotone mapping such that

$$ \biggl\langle v_{t_{0}}-v_{t}, \frac{v_{t}-u_{t}}{r_{t}}- \frac{v_{t_{0}}-u _{t_{0}}}{r_{t_{0}}}\biggr\rangle \geq 0, $$

and hence

3.20 $$ \biggl\langle v_{t}-v_{t_{0}},v_{t_{0}}-v_{t}+v_{t}-u_{t_{0}}- \frac{r_{t _{0}}}{r_{t}}(v_{t}-u_{t})\biggr\rangle \geq 0. $$

Taking into account that $\liminf_{t\to 0}r_{t}>0$, without loss of generality, we may assume that $r_{t}>b>0$ $\forall t\in (0, \min \{1, \frac{2-\bar{ \gamma }}{\tau -\gamma l}\})$ for some $b>0$. Then from (3.20) we have

$$\begin{aligned} \Vert v_{t}-v_{t_{0}} \Vert ^{2} &\leq \biggl\langle v_{t}-v_{t_{0}}, v_{t}-u_{t}+u _{t}-u_{t_{0}}-\frac{r_{t_{0}}}{r_{t}}(v_{t}-u_{t}) \biggr\rangle \\ &=\biggl\langle v_{t}-v_{t_{0}}, u_{t}-u_{t_{0}}+ \biggl(1-\frac{r_{t_{0}}}{r_{t}}\biggr) (v _{t}-u_{t})\biggr\rangle \\ &\leq \Vert v_{t}-v_{t_{0}} \Vert \biggl\Vert u_{t}-u_{t_{0}}+\biggl(1-\frac{r_{t_{0}}}{r_{t}}\biggr) (v _{t}-u_{t}) \biggr\Vert \\ &\leq \Vert v_{t}-v_{t_{0}} \Vert \biggl\{ \Vert u_{t}-u_{t_{0}} \Vert + \biggl\vert 1-\frac{r_{t_{0}}}{r _{t}} \biggr\vert \Vert v_{t}-u_{t} \Vert \biggr\} , \end{aligned}$$

which immediately yields

3.21 $$\begin{aligned} \Vert v_{t}-v_{t_{0}} \Vert &\leq \Vert u_{t}-u_{t_{0}} \Vert +\frac{1}{r_{t}} \vert r_{t}-r _{t_{0}} \vert \Vert v_{t}-u_{t} \Vert \\ &\leq \Vert u_{t}-u_{t_{0}} \Vert +\frac{\tilde{L}_{1}}{b} \vert r_{t}-r_{t_{0}} \vert , \end{aligned}$$

where $\tilde{L}_{1}=\sup \{\Vert v_{t}-u_{t}\Vert : t\in (0,\min \{1, \frac{2-\bar{ \gamma }}{\tau -\gamma l}\})\}$.

Also, taking into account that $\lim_{t\to 0}\lambda_{t}=\lambda >0$ and $\lim_{t\to 0}\nu_{t}=\nu >0$, without loss of generality, we may assume that $\min \{\lambda_{t}, \nu_{t}\}>a>0$ $\forall t\in (0,\min \{1, \frac{2-\bar{ \gamma }}{\tau -\gamma l}\})$ for some $a>0$. Since $z_{t}=F_{1, \lambda_{t}}y_{t}$ and $z_{t_{0}}=F_{1, \lambda_{t_{0}}}y_{t_{0}}$, where $y_{t}=F_{2, \nu_{t}}x_{t}$ and $y_{t_{0}}=F_{2,\nu_{t_{0}}}x _{t_{0}}$ for $t, t_{0}\in (0, \min \{1, \frac{2-\bar{\gamma }}{ \tau -\gamma l}\})$, by using arguments similar to those of (3.21), we get

3.22 $$ \Vert z_{t}-z_{t_{0}} \Vert \leq \Vert y_{t}-y_{t_{0}} \Vert +\frac{1}{a} \vert \lambda_{t}- \lambda_{t_{0}} \vert \tilde{L}_{2} $$

and

3.23 $$ \Vert y_{t}-y_{t_{0}} \Vert \leq \Vert x_{t}-x_{t_{0}} \Vert +\frac{1}{a} \vert \nu_{t}- \nu_{t_{0}} \vert \tilde{L}_{2}, $$

where $\tilde{L}_{2}=\sup \{\Vert z_{t}-y_{t}\Vert +\Vert y_{t}-x_{t}\Vert : t\in (0, \min \{1, \frac{2-\bar{\gamma }}{\tau -\gamma l}\})\}$. Substituting (3.23) for (3.22), we obtain

3.24 $$ \Vert z_{t}-z_{t_{0}} \Vert \leq \Vert x_{t}-x_{t_{0}} \Vert +\frac{\tilde{L}_{2}}{a}\bigl( \vert \lambda_{t}-\lambda_{t_{0}} \vert + \vert \nu_{t}-\nu_{t_{0}} \vert \bigr). $$

In the meantime, by Proposition 2.1(ii), (v) and Proposition 2.2, we deduce that

3.25 $$\begin{aligned} \Vert u_{t}-u_{t_{0}} \Vert &= \bigl\Vert {{\varDelta}}^{N}_{t}z_{t}-{\varDelta}^{N} _{t_{0}}z_{t_{0}} \bigr\Vert \\ &= \bigl\Vert T^{({\varTheta }_{N}, \varphi_{N})}_{r_{N, t}}(I-r_{N,t}B_{N}) {\varDelta}^{N-1}_{t}z_{t} -T^{({\varTheta }_{N}, \varphi_{N})} _{r_{N,t_{0}}}(I-r_{N, t_{0}}B_{N}){\varDelta}^{N-1}_{t_{0}}z_{t _{0}} \bigr\Vert \\ &\leq \bigl\Vert T^{({\varTheta }_{N},\varphi_{N})}_{r_{N,t}}(I-r_{N,t}B_{N}) {\varDelta}^{N-1}_{t}z_{t} -T^{({\varTheta }_{N}, \varphi_{N})} _{r_{N,t_{0}}}(I-r_{N, t_{0}}B_{N}){\varDelta}^{N-1}_{t}z_{t} \bigr\Vert \\ &\quad{}+ \bigl\Vert T^{({\varTheta }_{N},\varphi_{N})}_{r_{N,t_{0}}}(I-r_{N,t_{0}}B _{N}){\varDelta}^{N-1}_{t}z_{t} -T^{({\varTheta }_{N}, \varphi_{N})} _{r_{N,t_{0}}}(I-r_{N, t_{0}}B_{N}){\varDelta}^{N-1}_{t_{0}}z_{t _{0}} \bigr\Vert \\ &\leq \bigl\Vert T^{({\varTheta }_{N},\varphi_{N})}_{r_{N,t}}(I-r_{N,t}B_{N}) {\varDelta}^{N-1}_{t}z_{t} -T^{({\varTheta }_{N}, \varphi_{N})} _{r_{N,t_{0}}}(I-r_{N, t}B_{N}){\varDelta}^{N-1}_{t}z_{t} \bigr\Vert \\ & \quad{}+ \bigl\Vert T^{({\varTheta }_{N},\varphi_{N})}_{r_{N,t_{0}}}(I-r_{N,t}B_{N}) {\varDelta}^{N-1}_{t}z_{t} -T^{({\varTheta }_{N}, \varphi_{N})} _{r_{N,t_{0}}}(I-r_{N, t_{0}}B_{N}){\varDelta}^{N-1}_{t}z_{t} \bigr\Vert \\ &\quad{}+ \bigl\Vert (I-r_{N,t_{0}}B_{N}){\varDelta}^{N-1}_{t}z_{t}-(I-r_{N, t_{0}}B _{N}){\varDelta}^{N-1}_{t_{0}}z_{t_{0}} \bigr\Vert \\ &\leq \frac{ \vert r_{N, t}-r_{N,t_{0}} \vert }{r_{N, t}} \bigl\Vert T^{({\varTheta }_{N}, \varphi_{N})}_{r_{N,t}}(I-r_{N, t}B_{N}) {\varDelta}^{N-1}_{t}z_{t} -(I-r _{N,t}B_{N}){\varDelta}^{N-1}_{t}z_{t} \bigr\Vert \\ &\quad{}+ \vert r_{N,t}-r_{N, t_{0}} \vert \bigl\Vert B_{N}{{\varDelta}}^{N-1}_{t}z_{t} \bigr\Vert + \bigl\Vert {{\varDelta}}^{N-1}_{t}z_{t}- {\varDelta}^{N-1}_{t_{0}}z_{t_{0}} \bigr\Vert \\ &= \vert r_{N,t}-r_{N, t_{0}} \vert \biggl[ \bigl\Vert B_{N}{{\varDelta}}^{N-1}_{t}z_{t} \bigr\Vert +\frac{1}{r _{N, t}} \bigl\Vert T^{({\varTheta }_{N},\varphi_{N})}_{r_{N, t}} (I-r_{N,t}B _{N}){\varDelta}^{N-1}_{t}z_{t} \\ &\quad{}-(I-r_{N, t}B_{N}){\varDelta}^{N-1}_{t}z_{t} \bigr\Vert \biggr]+ \bigl\Vert {{\varDelta}}^{N-1} _{t}z_{t}-{\varDelta}^{N-1}_{t_{0}}z_{t_{0}} \bigr\Vert \\ &\leq \cdots \\ &\leq \vert r_{N, t}-r_{N, t_{0}} \vert \biggl[ \bigl\Vert B_{N}{{\varDelta}}^{N-1}_{t}z_{t} \bigr\Vert +\frac{1}{r _{N, t}} \bigl\Vert T^{({\varTheta }_{N}, \varphi_{N})}_{r_{N, t}} (I-r_{N, t}B _{N}){\varDelta}^{N-1}_{t}z_{t} \\ &\quad{}-(I-r_{N, t}B_{N}){\varDelta}^{N-1}_{t}z_{t} \bigr\Vert \biggr]+\cdots + \vert r_{1, t}-r _{1,t_{0}} \vert \biggl[ \bigl\Vert B_{1}{{\varDelta}}^{0}_{t}z_{t} \bigr\Vert \\ &\quad{}+\frac{1}{r_{1, t}} \bigl\Vert T^{({\varTheta }_{1}, \varphi_{1})}_{r_{1, t}}(I-r _{1, t}B_{1}){\varDelta}^{0}_{t}z_{t}- (I-r_{1, t}B_{1}){\varDelta}^{0}_{t}z_{t} \bigr\Vert \biggr]+ \bigl\Vert {{\varDelta}}^{0}_{t}z_{t}- {\varDelta}^{0}_{t _{0}}z_{t_{0}} \bigr\Vert \\ &\leq \widetilde{L}_{3}{ \sum^{N}_{i=1}} \vert r_{i, t}-r_{i, t_{0}} \vert + \Vert z_{t}-z_{t_{0}} \Vert , \end{aligned}$$

where

$$ \begin{aligned} & \sup_{t\in (0,\min \{1, \frac{2-\bar{\gamma }}{\tau -\gamma l}\})}\Biggl\{ \sum^{N}_{i=1} \biggl[ \bigl\Vert B_{i}{{\varDelta}}^{i-1}_{t}z_{t} \bigr\Vert + \frac{1}{r_{i,t}} \bigl\Vert T^{({\varTheta }_{i}, \varphi_{i})}_{r_{i, t}}(I-r _{i, t}B_{i}){\varDelta}^{i-1}_{t}z_{t} -(I-r_{i, t}B_{i}){\varDelta}^{i-1}_{t}z_{t} \bigr\Vert \biggr]\Biggr\} \\&\quad \leq \widetilde{L}_{3} \end{aligned} $$

for some $\widetilde{L}_{3}>0$. This together with (3.21) and (3.24) implies that

$$\begin{aligned} \Vert v_{t}-v_{t_{0}} \Vert &\leq \Vert u_{t}-u_{t_{0}} \Vert +\frac{\tilde{L}_{1}}{b} \vert r _{t}-r_{t_{0}} \vert \\ &\leq \widetilde{L}_{3}{ \sum^{N}_{i=1}} \vert r_{i, t}-r_{i, t_{0}} \vert + \Vert z_{t}-z_{t_{0}} \Vert +\frac{ \tilde{L}_{1}}{b} \vert r_{t}-r_{t_{0}} \vert \\ &\leq \widetilde{L}_{3}{ \sum^{N}_{i=1}} \vert r_{i, t}-r_{i, t_{0}} \vert + \Vert x_{t}-x_{t_{0}} \Vert +\frac{ \tilde{L}_{2}}{a}\bigl( \vert \lambda_{t}- \lambda_{t_{0}} \vert + \vert \nu_{t}-\nu_{t_{0}} \vert \bigr)+\frac{ \tilde{L}_{1}}{b} \vert r_{t}-r_{t_{0}} \vert \\ &\leq \Vert x_{t}-x_{t_{0}} \Vert +\biggl( \frac{\tilde{L}_{1}}{b}+\frac{\tilde{L} _{2}}{a}\biggr) \bigl( \vert \lambda_{t}- \lambda_{t_{0}} \vert + \vert \nu_{t}-\nu_{t_{0}} \vert + \vert r_{t}-r _{t_{0}} \vert \bigr) \\ &\quad{} + \widetilde{L}_{3}{ \sum^{N}_{i=1}} \vert r_{i,t}-r_{i,t_{0}} \vert . \end{aligned}$$

Taking into account that both $\theta_{t_{0}}\in (0, \min \{ \frac{1}{2}, \Vert A\Vert ^{-1}\})$ and $0\leq \gamma l<\tau =1- \sqrt{1- \mu (2\eta -\mu \rho^{2})}$ imply

$$ 0< 1-\theta_{t_{0}}(\bar{\gamma }-1+t_{0}\tau )< 1, $$

we calculate from (3.1)

$$\begin{aligned} \Vert x_{t}-x_{t_{0}} \Vert &\leq \bigl\Vert (I- \theta_{t}A)T_{r_{t}}{{\varDelta}}^{N} _{t}R_{t}x_{t}+\theta_{t}\bigl(t \gamma Vx_{t}+(I-t\mu G)T_{r_{t}}{{\varDelta}}^{N}_{t}R_{t}x_{t}\bigr) \\ &\quad{}-(I-\theta_{t_{0}}A)T_{r_{t_{0}}}{{\varDelta}}^{N}_{t_{0}}R_{t_{0}}x _{t_{0}}+ \theta_{t_{0}}\bigl(t_{0}\gamma Vx_{t_{0}}+(I-t_{0} \mu G)T_{r_{t _{0}}}{{\varDelta}}^{N}_{t_{0}}R_{t_{0}}x_{t_{0}} \bigr) \bigr\Vert \\ &= \bigl\Vert (I-\theta_{t}A)v_{t}+\theta_{t} \bigl(t\gamma Vx_{t}+(I-t\mu G)v_{t}\bigr)-(I- \theta_{t_{0}}A)v_{t_{0}} \\ &\quad{}+\theta_{t_{0}}\bigl(t_{0} \gamma Vx_{t_{0}}+(I-t _{0}\mu G)v_{t_{0}}\bigr) \bigr\Vert \\ &\leq \bigl\Vert (I-\theta_{t}A)v_{t}-(I- \theta_{t_{0}}A)v_{t} \bigr\Vert + \bigl\Vert (I- \theta_{t_{0}}A)v_{t}-(I-\theta_{t_{0}}A)v_{t_{0}} \bigr\Vert \\ &\quad{}+ \vert \theta_{t}-\theta_{t_{0}} \vert \bigl\Vert t\gamma Vx_{t}+(I-t\mu G)v_{t} \bigr\Vert + \theta_{t_{0}} \bigl\Vert \bigl[t\gamma Vx_{t}+(I-t\mu G)v_{t}\bigr] \\ &\quad{}-\bigl[t_{0}\gamma Vx_{t_{0}}+(I-t_{0}\mu G)v_{t_{0}}\bigr] \bigr\Vert \\ &\leq \vert \theta_{t}-\theta_{t_{0}} \vert \Vert A \Vert \Vert v_{t} \Vert +(1-\theta_{t_{0}}\bar{ \gamma }) \Vert v_{t}-v_{t_{0}} \Vert + \vert \theta_{t}- \theta_{t_{0}} \vert \bigl\Vert t\gamma Vx _{t}+(I-t\mu G)v_{t} \bigr\Vert \\ &\quad{}+\theta_{t_{0}} \bigl\Vert (t-t_{0})\gamma Vx_{t}+t_{0}\gamma (Vx_{t}-Vx_{t_{0}})-(t-t _{0})\mu Gv_{t}+(I-t_{0}\mu G)v_{t} \\ &\quad{}-(I-t_{0} \mu G)v_{t_{0}} \bigr\Vert \\ &\leq \vert \theta_{t}-\theta_{t_{0}} \vert \Vert A \Vert \Vert v_{t} \Vert +(1-\theta_{t_{0}}\bar{ \gamma }) \Vert v_{t}-v_{t_{0}} \Vert + \vert \theta_{t}- \theta_{t_{0}} \vert \bigl[ \Vert v_{t} \Vert \\ &\quad{}+t\bigl( \gamma \Vert Vx_{t} \Vert +\mu \Vert Gv_{t} \Vert \bigr)\bigr] \\ &\quad{}+\theta_{t_{0}}\bigl[\bigl(\gamma \Vert Vx_{t} \Vert +\mu \Vert Gv_{t} \Vert \bigr) \vert t-t_{0} \vert +t_{0} \gamma l \Vert x_{t}-x_{t_{0}} \Vert +(1-t_{0}\tau ) \Vert v_{t}-v_{t_{0}} \Vert \bigr] \\ &\leq \vert \theta_{t}-\theta_{t_{0}} \vert \bigl[ \Vert v_{t} \Vert + \Vert A \Vert \Vert v_{t} \Vert + \gamma \Vert Vx _{t} \Vert +\mu \Vert Gv_{t} \Vert \bigr]+\theta_{t_{0}}t_{0}\gamma l \Vert x_{t}-x_{t_{0}} \Vert \\ &\quad{}+\bigl[1-\theta_{t_{0}}(\bar{\gamma }-1+t_{0}\tau ) \bigr] \\ &\quad{}\times\Biggl\{ \Vert x_{t}-x_{t_{0}} \Vert +\biggl( \frac{ \tilde{L}_{1}}{b}+\frac{\tilde{L}_{2}}{a}\biggr) \bigl( \vert \lambda_{t}- \lambda_{t _{0}} \vert + \vert \nu_{t}-\nu_{t_{0}} \vert + \vert r_{t}-r_{t_{0}} \vert \bigr) \\ &\quad{}+\widetilde{L}_{3}{ \sum^{N}_{i=1}} \vert r_{i,t}-r_{i,t_{0}} \vert \Biggr\} +\theta_{t_{0}} \bigl(\gamma \Vert Vx_{t} \Vert +\mu \Vert Gv_{t} \Vert \bigr) \vert t-t_{0} \vert \\ &= \vert \theta_{t}-\theta_{t_{0}} \vert \bigl[ \Vert v_{t} \Vert + \Vert A \Vert \Vert v_{t} \Vert + \gamma \Vert Vx _{t} \Vert +\mu \Vert Gv_{t} \Vert \bigr] \\ &\quad{} +\bigl[1-\theta_{t_{0}}(\bar{\gamma }-1+t_{0}( \tau - \gamma l)\bigr] \Vert x_{t}-x_{t_{0}} \Vert \\ &\quad{}+\bigl[1-\theta_{t_{0}}(\bar{\gamma }-1+t_{0}\tau ) \bigr]\Biggl\{ \biggl( \frac{\tilde{L}_{1}}{b}+\frac{\tilde{L}_{2}}{a}\biggr) \bigl( \vert \lambda_{t}- \lambda_{t_{0}} \vert + \vert \nu_{t}-\nu_{t_{0}} \vert + \vert r_{t}-r_{t_{0}} \vert \bigr) \\ &\quad{}+\widetilde{L}_{3}{ \sum^{N}_{i=1}} \vert r_{i,t}-r_{i,t_{0}} \vert \Biggr\} +\theta_{t_{0}} \bigl(\gamma \Vert Vx_{t} \Vert +\mu \Vert Gv_{t} \Vert \bigr) \vert t-t_{0} \vert . \end{aligned}$$

This immediately implies that

$$\begin{aligned} \Vert x_{t}-x_{t_{0}} \Vert &\leq \frac{ \Vert v_{t} \Vert + \Vert A \Vert \Vert v_{t} \Vert +\gamma \Vert Vx _{t} \Vert +\mu \Vert Gv_{t} \Vert }{\theta_{t_{0}}(\bar{\gamma }-1+t_{0}(\tau - \gamma l)} \vert \theta_{t}-\theta_{t_{0}} \vert +\frac{\gamma \Vert Vx_{t} \Vert +\mu \Vert Gv _{t} \Vert }{\bar{\gamma }-1+t_{0}(\tau -\gamma l)} \vert t-t_{0} \vert \\ &\quad{}+\frac{1-\theta_{t_{0}}(\bar{\gamma }-1+t_{0}\tau )}{\theta_{t_{0}}(\bar{ \gamma }-1+t_{0}(\tau -\gamma l)}\Biggl\{ \biggl(\frac{\tilde{L}_{1}}{b}+\frac{ \tilde{L}_{2}}{a} \biggr) \bigl( \vert \lambda_{t}-\lambda_{t_{0}} \vert + \vert \nu_{t}-\nu_{t_{0}} \vert + \vert r _{t}-r_{t_{0}} \vert \bigr) \\ &\quad{}+\widetilde{L}_{3}{ \sum^{N}_{i=1}} \vert r_{i, t}-r_{i, t_{0}} \vert \Biggr\} . \end{aligned}$$

Since $\theta_{t}:(0, \min \{1, \frac{2-\bar{\gamma }}{\tau -\gamma l}\})\to (0,\min \{\frac{1}{2},\Vert A \Vert ^{-1}\})$ is locally Lipschitzian, $r_{t},\lambda_{t},\nu_{t}:(0, \min \{1, \frac{2-\bar{\gamma }}{\tau -\gamma l}\})\to (0, \infty )$ are locally Lipschitzian, and $r_{i, t}: (0,\min \{1,\frac{2-\bar{\gamma }}{\tau -\gamma l}\})\to [c_{i},d_{i}]$ is locally Lipschitzian for each $i=1, 2,\ldots, N$, we deduce that $x_{t}:(0, \min \{1, \frac{2-\bar{ \gamma }}{\tau -\gamma l}\})\to H$ is locally Lipschitzian.

(iv) From the last inequality in (iii), the desired result follows immediately. □

We prove the following strong convergence theorem for the net $\{x_{t}\}$ as $t\to 0$, which guarantees the existence of solutions of the variational inequality (3.2).

### Theorem 3.2

*Let the net* $\{x_{t}\}$ *be defined via* (3.1). *If* $\lim_{t\to 0}\theta_{t}=0$, *then* $x_{t}$ *converges strongly to* $\widetilde{x}\in {{\varOmega }}$ *as* $t\to 0$, *which solves VI* (3.2). *Equivalently*, *we have* $P_{{\varOmega }} (2I-A)\widetilde{x}= \widetilde{x}$.

### Proof

We first note that the uniqueness of a solution of VI (3.2) is a consequence of the strong monotonicity of $A-I$ (due to Lemma 2.4). See [2, 4, 5] for this fact.

Next, we prove that $x_{t}\to \widetilde{x}$ as $t\to 0$. For simplicity, let $v_{t}=T_{r_{t}}u_{t}$, $u_{t}={\varDelta}^{N}_{t}z _{t}$, $y_{t}= F_{2,\nu_{t}}x_{t}$, and $z_{t}=R_{t}x_{t}=F_{1,\lambda _{t}}y_{t}$. For any given $p\in {{\varOmega }}$, we observe that $T_{r_{t}}p=p$, ${{\varDelta}}^{N}_{t}p =p$, and $R_{t}p=p$. From (3.1), we write

$$\begin{aligned} x_{t}-p&=x_{t}-w_{t}+w_{t}-p=x_{t}-w_{t}+(I- \theta_{t}A)v_{t}+\theta _{t}\bigl(t\gamma Vx_{t}+(I-t\mu G)v_{t}\bigr)-p \\ &=x_{t}-w_{t}+(I-\theta_{t}A) (v_{t}-p)+\theta_{t}\bigl[t(\gamma Vx_{t}- \mu Gp)+(I-t\mu G)v_{t}-(I-t\mu G)p\bigr] \\ &\quad{} +\theta_{t}(I-A)p, \end{aligned}$$

where $w_{t}=(I-\theta_{t}A)v_{t}+\theta_{t}(t\gamma Vx_{t}+(I-t \mu G)v_{t})$. In terms of (2.1) and (3.5), we have

$$\begin{aligned} \Vert x_{t}-p \Vert ^{2} &=\langle x_{t}-w_{t}, x_{t}-p\rangle +\bigl\langle (I- \theta_{t}A) (v_{t}-p), x_{t}-p\bigr\rangle +\theta_{t} \bigl[t\langle \gamma Vx _{t}-\mu Gp,x_{t}-p\rangle \\ &\quad{}+\bigl\langle (I-t\mu G)v_{t}-(I-t\mu G)p,x_{t}-p \bigr\rangle \bigr]+\theta_{t} \bigl\langle (I-A)p, x_{t}-p \bigr\rangle \\ &\leq (1-\theta_{t}\bar{\gamma }) \Vert x_{t}-p \Vert ^{2}+\theta_{t}\bigl[(1-t \tau ) \Vert x_{t}-p \Vert ^{2}+t\gamma l \Vert x_{t}-p \Vert ^{2} \\ &\quad{}+t\bigl\langle (\gamma V-\mu G)p,x_{t}-p\bigr\rangle \bigr]+ \theta_{t}\bigl\langle (I-A)p,x _{t}-p\bigr\rangle \\ &=\bigl[1-\theta_{t}\bigl(\bar{\gamma }-1+t(\tau -\gamma l)\bigr) \bigr] \Vert x_{t}-p \Vert ^{2}+ \theta_{t} \bigl(t\bigl\langle (\gamma V-\mu G)p,x_{t}-p\bigr\rangle \\ &\quad{}+\bigl\langle (I-A)p,x _{t}-p\bigr\rangle \bigr). \end{aligned}$$

Therefore,

3.26 $$ \Vert x_{t}-p \Vert ^{2}\leq \frac{1}{\bar{\gamma }-1+t(\tau -\gamma l)}\bigl(t \bigl\langle (\gamma V-\mu G)p,x_{t}-p\bigr\rangle +\bigl\langle (I-A)p,x_{t}-p \bigr\rangle \bigr). $$

Since $\{x_{t}\}$ is bounded as $t\to 0$ (due to Theorem 3.1(i)), there exists a subsequence $\{t_{n}\}$ in $(0,\min \{1, \frac{2-\bar{\gamma }}{\tau -\gamma l}\})$ such that $t_{n}\to 0$ and $x_{t_{n}}\rightharpoonup x^{*}$. We first show that $x^{*}\in {{\varOmega }}$. To this end, we divide its proof into four steps.

*Step 1.* We claim that $\lim_{n\to \infty }\Vert x_{t_{n}}-z_{t_{n}} \Vert =0$, $\lim_{n\to \infty }\Vert z_{t_{n}}-u_{t_{n}}\Vert =0$, and $\lim_{n\to \infty }\Vert u_{t_{n}}-v_{t_{n}}\Vert =0$, where $z_{t_{n}}=R_{t _{n}}x_{t_{n}}$, $u_{t_{n}}={\varDelta}^{N}_{t_{n}}z_{t_{n}}$, and $v_{t_{n}}=T_{r_{t_{n}}}u_{t_{n}}$. Indeed, according to (3.9), (3.12), and (3.14) in the proof of Theorem 3.1, we obtain the assertion.

*Step 2.* We claim that $x^{*}\in \operatorname {Fix}(T)$. In fact, from the definition of $v_{t_{n}}=T_{r_{t_{n}}}u_{t_{n}}$, we have

3.27 $$ \bigl\langle y-v_{t_{n}},(I-T)v_{t_{n}}\bigr\rangle +\biggl\langle y-v_{t_{n}},\frac{v _{t_{n}}-u_{t_{n}}}{r_{t_{n}}}\biggr\rangle \geq 0,\quad \forall y \in C. $$

Set $w_{t}=tv+(1-t)x^{*}$ for all $t\in (0,1]$ and $v\in C$. Then $w_{t}\in C$. From (3.27) it follows that

3.28 $$\begin{aligned} \bigl\langle w_{t}-v_{t_{n}},(I-T)w_{t}\bigr\rangle &\geq \bigl\langle w_{t}-v_{t _{n}},(I-T)w_{t} \bigr\rangle -\bigl\langle w_{t}-v_{t_{n}},(I-T)v_{t_{n}} \bigr\rangle -\biggl\langle w_{t}-v_{t_{n}},\frac{v_{t_{n}}-u_{t_{n}}}{r_{t_{n}}} \biggr\rangle \\ &=\bigl\langle w_{t}-v_{t_{n}},(I-T)w_{t}-(I-T)v_{t_{n}} \bigr\rangle -\biggl\langle w _{t}-v_{t_{n}},\frac{v_{t_{n}}-u_{t_{n}}}{r_{t_{n}}} \biggr\rangle . \end{aligned}$$

By Step 1, we have $\frac{v_{t_{n}}-u_{t_{n}}}{r_{t_{n}}}\to 0$ as $n\to \infty $. Moreover, since $x_{t_{n}}\rightharpoonup x^{*}$, by Step 1 we have $v_{t_{n}}\rightharpoonup x^{*}$. Since $I-T$ is monotone, we also have that $\langle w_{t}-v_{t_{n}}, (I-T)w_{t}-(I-T)v _{t_{n}}\rangle \geq 0$. Thus, from (3.28) it follows that

$$ 0\leq \lim_{n\to \infty }\bigl\langle w_{t}-v_{t_{n}}, (I-T)w_{t}\bigr\rangle = \bigl\langle w_{t}-x^{*}, (I-T)w_{t}\bigr\rangle , $$

and hence

$$ \bigl\langle v-x^{*},(I-T)w_{t}\bigr\rangle \geq 0,\quad \forall v\in C. $$

Letting $t\to 0$, we know from the continuity of $I-T$ that

$$ \bigl\langle v-x^{*}, (I-T)x^{*}\bigr\rangle \geq 0,\quad \forall v\in C. $$

Putting $v=Tx^{*}$, we get $\Vert (I-T)x^{*}\Vert ^{2}=0$, which leads to $x^{*}\in \operatorname {Fix}(T)$.

*Step 3.* We claim that $x^{*}\in \operatorname {GSVI}(C, F_{1}, F_{2})$. Indeed, note that $\lim_{t\to 0}\lambda_{t}=\lambda >0$ and $\lim_{t\to 0}\nu_{t}=\nu >0$. For each $x\in C$, we put $x(t):=F_{1, \lambda_{t}}x$, $x(0):=F_{1,\lambda }x$, $y(t):=F_{2, \nu_{t}}x$, and $y(0):=F_{2,\nu }x$. Then, by Lemma 1.1, we have $\operatorname {GSVI}(C, F_{1}, F_{2})=\operatorname {Fix}(R)$, where $R=F_{1,\lambda }F_{2,\nu }$ and *R* is nonexpansive. Moreover, it is easy to see that

3.29 $$ \bigl\langle y-x(t), F_{1}x(t)\bigr\rangle +\frac{1}{\lambda_{t}}\bigl\langle y-x(t), x(t)-x\bigr\rangle \geq 0,\quad \forall y\in C, $$

and

3.30 $$ \bigl\langle y-x(0), F_{1}x(0)\bigr\rangle +\frac{1}{\lambda }\bigl\langle y-x(0), x(0)-x \bigr\rangle \geq 0,\quad \forall y\in C. $$

Putting $y=x(0)$ in (3.29) and $y=x(t)$ in (3.30), we obtain

3.31 $$ \bigl\langle x(0)-x(t), F_{1}x(t)\bigr\rangle +\frac{1}{\lambda_{t}} \bigl\langle x(0)-x(t), x(t)-x \bigr\rangle \geq 0 $$

and

3.32 $$ \bigl\langle x(t)-x(0), F_{1}x(0)\bigr\rangle +\frac{1}{\lambda } \bigl\langle x(t)-x(0), x(0)-x\bigr\rangle \geq 0. $$

Adding up (3.31) and (3.32), we have

$$ -\bigl\langle x(t)-x(0), F_{1}x(t)-F_{1}x(0)\bigr\rangle + \biggl\langle x(0)-x(t), \frac{x(t)-x}{ \lambda_{t}}-\frac{x(0)-x}{\lambda }\biggr\rangle \geq 0. $$

Since $F_{1}$ is a monotone mapping, we know that

$$ \biggl\langle x(0)-x(t), \frac{x(t)-x}{\lambda_{t}}-\frac{x(0)-x}{\lambda } \biggr\rangle \geq 0, $$

and hence

$$ \biggl\langle x(t)-x(0), x(0)-x(t)+x(t)-x-\frac{\lambda }{\lambda_{t}}\bigl(x(t)-x\bigr) \biggr\rangle \geq 0. $$

So it follows that

$$\begin{aligned} \bigl\Vert x(t)-x(0) \bigr\Vert ^{2} &\leq \biggl\langle x(t)-x(0), x(t)-x-\frac{\lambda }{ \lambda_{t}}\bigl(x(t)-x\bigr)\biggr\rangle \\ &=\biggl\langle x(t)-x(0), \biggl(1-\frac{\lambda }{\lambda_{t}}\biggr) \bigl(x(t)-x\bigr) \biggr\rangle \\ &\leq \bigl\Vert x(t)-x(0) \bigr\Vert \cdot \frac{ \vert \lambda_{t}-\lambda \vert }{\lambda_{t}} \bigl\Vert x(t)-x \bigr\Vert , \end{aligned}$$

which immediately yields

3.33 $$ \Vert F_{1,\lambda_{t}}x-F_{1,\lambda }x \Vert \leq \frac{ \vert \lambda_{t}-\lambda \vert }{\lambda_{t}} \Vert F_{1, \lambda_{t}}x-x \Vert . $$

By using arguments similar to those of (3.33), we have

3.34 $$ \Vert F_{2,\nu_{t}}x-F_{2,\nu }x \Vert \leq \frac{ \vert \nu_{t}-\nu \vert }{\nu_{t}} \Vert F _{2, \nu_{t}}x-x \Vert . $$

Now, putting $t=t_{n}$, $x=F_{2,\nu }x_{t_{n}}$ in (3.33), and $t=t_{n}$, $x=x_{t_{n}}$ in (3.34), respectively, we deduce that

$$ \Vert F_{1,\lambda_{t_{n}}}F_{2,\nu }x_{t_{n}}-F_{1,\lambda }F_{2,\nu }x _{t_{n}} \Vert \leq \frac{ \vert \lambda_{t_{n}}-\lambda \vert }{\lambda_{t_{n}}} \Vert F _{1, \lambda_{t_{n}}}F_{2, \nu }x_{t_{n}}-F_{2, \nu }x_{t_{n}} \Vert $$

and

$$ \Vert F_{2,\nu_{t_{n}}}x_{t_{n}}-F_{2, \nu }x_{t_{n}} \Vert \leq \frac{ \vert \nu _{t_{n}}-\nu \vert }{\nu_{t_{n}}} \Vert F_{2, \nu_{t_{n}}}x_{t_{n}}-x_{t_{n}} \Vert . $$

Since $\lim_{n\to \infty }\lambda_{t_{n}}=\lambda >0$ and $\lim_{n\to \infty }\nu_{t_{n}}=\nu >0$, it follows from the last two inequalities that

3.35 $$ \lim_{n\to \infty } \Vert F_{1, \lambda_{t_{n}}}F_{2, \nu }x_{t_{n}}-F_{1, \lambda }F_{2,\nu }x_{t_{n}} \Vert =\lim_{n\to \infty } \Vert F_{2, \nu_{t_{n}}}x _{t_{n}}-F_{2, \nu }x_{t_{n}} \Vert =0. $$

Also, we observe that

3.36 $$\begin{aligned} & \Vert Rx_{t_{n}}-x_{t_{n}} \Vert \\ &\quad \leq \Vert F_{1,\lambda }F_{2, \nu }x_{t_{n}}-F_{1, \lambda_{t_{n}}}F _{2, \nu }x_{t_{n}} \Vert + \Vert F_{1,\lambda_{t_{n}}}F_{2, \nu }x_{t_{n}}-F _{1, \lambda_{t_{n}}}F_{2, \nu_{t_{n}}}x_{t_{n}} \Vert \\ &\quad\quad{} + \Vert F_{1,\lambda _{t_{n}}}F_{2, \nu_{t_{n}}}x_{t_{n}}-x_{t_{n}} \Vert \\ &\quad \leq \Vert F_{1,\lambda }F_{2, \nu }x_{t_{n}}-F_{1,\lambda_{t_{n}}}F_{2, \nu }x_{t_{n}} \Vert + \Vert F_{2,\nu }x_{t_{n}}-F_{2, \nu_{t_{n}}}x_{t_{n}} \Vert + \Vert F_{1,\lambda_{t_{n}}}F_{2, \nu_{t_{n}}}x_{t_{n}}-x_{t_{n}} \Vert \\ &\quad = \Vert F_{1,\lambda }F_{2,\nu }x_{t_{n}}-F_{1,\lambda_{t_{n}}}F_{2, \nu }x_{t_{n}} \Vert + \Vert F_{2,\nu }x_{t_{n}}-F_{2, \nu_{t_{n}}}x_{t_{n}} \Vert + \Vert R_{t_{n}}x_{t_{n}}-x_{t_{n}} \Vert . \end{aligned}$$

Since $R_{t_{n}}x_{t_{n}}-x_{t_{n}}\to 0$ (due to Step 1), from (3.35) and (3.36) we get

3.37 $$ \lim_{n\to \infty } \Vert Rx_{t_{n}}-x_{t_{n}} \Vert =0. $$

Taking into account that $x_{t_{n}}\rightharpoonup x^{*}$ and $x_{t_{n}}-Rx_{t_{n}}\to 0$ (due to (3.37)), from Lemma 2.3 we get $x^{*}=Rx^{*}$, that is, $x^{*}\in \operatorname {Fix}(R)=\operatorname {GSVI}(C,F_{1},F _{2})$.

*Step 4.* We claim that $x^{*}\in \bigcap^{N}_{i=1}\operatorname {GMEP}( {\varTheta }_{i}, \varphi_{i}, B_{i})$. In fact, since ${\varDelta}^{i}_{t_{n}}z_{t_{n}}=T^{({\varTheta }_{i}, \varphi_{i})}_{r_{i, t _{n}}}(I-r_{i, t_{n}}B_{i}){\varDelta}^{i-1}_{t_{n}}z_{t_{n}}$, for each $i=1, 2,\ldots, N$, we have

$$\begin{aligned} 0&\leq {{\varTheta }}_{i}\bigl({\varDelta}^{i}_{t_{n}}z_{t_{n}},y\bigr)+\varphi _{i}(y)-\varphi_{i}\bigl({\varDelta}^{i}_{t_{n}}z_{t_{n}}\bigr) \\ &\quad{}+\bigl\langle B_{i}{{\varDelta}}^{i-1}_{t_{n}}z_{t_{n}},y- {\varDelta} ^{i}_{t_{n}}z_{t_{n}}\bigr\rangle + \frac{1}{r_{i,t_{n}}}\bigl\langle y-{\varDelta}^{i}_{t_{n}}z_{t_{n}}, {\varDelta}^{i}_{t_{n}}z_{t_{n}}- {\varDelta}^{i-1} _{t_{n}}z_{t_{n}}\bigr\rangle . \end{aligned}$$

By (A2), we have

$$\begin{aligned} {{\varTheta }}_{i}\bigl(y,{\varDelta}^{i}_{t_{n}}z_{t_{n}} \bigr)&\leq \varphi _{i}(y)-\varphi_{i}\bigl({\varDelta}^{i}_{t_{n}}z_{t_{n}}\bigr) +\bigl\langle B _{i}{{\varDelta}}^{i-1}_{t_{n}}z_{t_{n}}, y-{\varDelta}^{i}_{t_{n}}z _{t_{n}}\bigr\rangle \\ &\quad{}+\frac{1}{r_{i, t_{n}}}\bigl\langle y-{\varDelta}^{i}_{t_{n}}z_{t_{n}}, {\varDelta}^{i}_{t_{n}}z_{t_{n}}- {\varDelta}^{i-1}_{t_{n}}z_{t _{n}}\bigr\rangle . \end{aligned}$$

Let $w_{t}=tv+(1-t)x^{*}$ for all $t\in (0,1]$ and $v\in C$. This implies that $w_{t}\in C$. Then we have

$$\begin{aligned} &\bigl\langle w_{t}-{\varDelta}^{i}_{t_{n}}z_{t_{n}},B_{i}w_{t} \bigr\rangle \\ &\quad \geq \varphi_{i}\bigl({\varDelta}^{i}_{t_{n}}z_{t_{n}} \bigr)-\varphi_{i}(w _{t})+\bigl\langle w_{t}- {\varDelta}^{i}_{t_{n}}z_{t_{n}},B_{i}w_{t} \bigr\rangle -\bigl\langle w_{t}-{\varDelta}^{i}_{t_{n}}z_{t_{n}},B_{i}{ {\varDelta}}^{i-1}_{t_{n}}z_{t_{n}}\bigr\rangle \\ &\quad \quad{}-\biggl\langle w_{t}-{\varDelta}^{i}_{t_{n}}z_{t_{n}},\frac{{\varDelta} ^{i}_{t_{n}}z_{t_{n}}- {\varDelta}^{i-1}_{t_{n}}z_{t_{n}}}{r_{i,t _{n}}}\biggr\rangle +{\varTheta }_{i}\bigl(w_{t},{\varDelta}^{i}_{t_{n}}z_{t _{n}}\bigr) \\ &\quad =\varphi_{i}\bigl({\varDelta}^{i}_{t_{n}}z_{t_{n}} \bigr)-\varphi_{i}(w_{t})+ \bigl\langle w_{t}- {\varDelta}^{i}_{t_{n}}z_{t_{n}},B_{i}w_{t}-B_{i} {{\varDelta}}^{i}_{t_{n}}z_{t_{n}}\bigr\rangle \\ &\quad \quad{}+\bigl\langle w_{t}-{\varDelta}^{i}_{t_{n}}z_{t_{n}},B_{j}{ {\varDelta}} ^{i}_{t_{n}}z_{t_{n}}-B_{i}{ {\varDelta}}^{i-1}_{t_{n}}z_{t_{n}}\bigr\rangle \\ &\quad \quad{}-\biggl\langle w_{t}-{\varDelta}^{i}_{t_{n}}z_{t_{n}},\frac{{\varDelta} ^{i}_{t_{n}}z_{t_{n}}- {\varDelta}^{i-1}_{t_{n}}z_{t_{n}}}{r_{i,t _{n}}}\biggr\rangle +{\varTheta }_{i}\bigl(w_{t},{\varDelta}^{i}_{t_{n}}z_{t _{n}}\bigr). \end{aligned}$$

By the same arguments as in the proof of Theorem 3.1, we have $\Vert B_{i}{{\varDelta}}^{i}_{t_{n}}z_{t_{n}}-B_{i}{{\varDelta}}^{i-1} _{t_{n}}z_{t_{n}}\Vert \to 0$ as $n\to \infty $. In the meantime, by the monotonicity of $B_{i}$, we obtain $\langle w_{t}-{\varDelta}^{i} _{t_{n}}z_{t_{n}},B_{i}w_{t}-B_{i}{{\varDelta}}^{i}_{t_{n}} z_{t_{n}} \rangle \geq 0$. Then by (A4) we get

$$ \bigl\langle w_{t}-x^{*},B_{i}w_{t} \bigr\rangle \geq \varphi_{i}\bigl(x^{*}\bigr)-\varphi _{i}(w_{t})+{\varTheta }_{i} \bigl(w_{t},x^{*}\bigr). $$

Utilizing (A1), (A4), and the last inequality, we obtain

$$\begin{aligned} 0&={\varTheta }_{i}(w_{t},w_{t})+ \varphi_{i}(w_{t})-\varphi_{i}(w _{t}) \\ &\leq t{\varTheta }_{i}(w_{t}, v)+(1-t){\varTheta }_{i}\bigl(w_{t}, x ^{*}\bigr)+t \varphi_{i}(v)+(1-t)\varphi_{i}\bigl(x^{*}\bigr)- \varphi_{i}(w_{t}) \\ &\leq t\bigl[{{\varTheta }}_{i}(w_{t}, v)+ \varphi_{i}(v)-\varphi_{i}(w_{t})\bigr]+(1-t) \bigl\langle w_{t}-x^{*}, B_{i}w_{t} \bigr\rangle \\ &=t\bigl[{{\varTheta }}_{i}(w_{t}, v)+ \varphi_{i}(v)-\varphi_{i}(w_{t})\bigr]+(1-t)t \bigl\langle v-x^{*}, B_{i}w_{t}\bigr\rangle , \end{aligned}$$

and hence

$$ 0\leq {{\varTheta }}_{i}(w_{t},v)+ \varphi_{i}(v)-\varphi_{i}(w_{t})+(1-t) \bigl\langle v-x^{*}, B_{i}w_{t}\bigr\rangle . $$

Letting $t\to 0$, we have, for each $v\in C$,

$$ 0\leq {{\varTheta }}_{i}\bigl(x^{*}, v\bigr)+ \varphi_{i}(v)-\varphi_{i}\bigl(x^{*}\bigr)+ \bigl\langle v-x^{*}, B_{i}x^{*}\bigr\rangle . $$

This implies that $x^{*}\in \operatorname {GMEP}({\varTheta }_{i},\varphi_{i}, B_{i})$ and hence $x^{*}\in \bigcap^{N}_{i=1}\operatorname {GMEP}({\varTheta } _{i}, \varphi_{i}, B_{i})$. This together with Steps 2 and 3 attains $x^{*}\in {{\varOmega }}$.

Finally, we show that $x^{*}$ is a solution of VI (3.2). In fact, putting $x_{t_{n}}$ in place of $x_{t}$ in (3.26) and taking the limit as $t_{n}\to 0$, we obtain

$$ \bigl\Vert x^{*}-p \bigr\Vert ^{2}\leq \frac{1}{\bar{\gamma }-1}\bigl\langle (I-A)p,x^{*}-p \bigr\rangle ,\quad \forall p\in {{\varOmega }}. $$

In particular, $x^{*}$ solves the following VI:

$$ x^{*}\in {{\varOmega }},\quad \bigl\langle (A-I)p, x^{*}-p\bigr\rangle \leq 0, \quad \forall p\in {{\varOmega }}, $$

or the equivalent dual variational inequality

$$ x^{*}\in {{\varOmega }},\quad \bigl\langle (A-I)x^{*}, x^{*}-p\bigr\rangle \leq 0,\quad \forall p\in {{\varOmega }}. $$

That is, $x^{*}\in {{\varOmega }}$ is a solution of VI (3.2). Hence $x^{*}=\widetilde{x}$ by uniqueness. In a summary, we have proven that each cluster point of $\{x_{t}\}$ (as $t\to 0$) equals *x̃*. Therefore $x_{t}\to \widetilde{x}$ as $t\to 0$. VI (3.2) can be rewritten as

$$ \bigl\langle (2I-A)\widetilde{x}-\widetilde{x},\widetilde{x}-p\bigr\rangle \geq 0,\quad \forall p\in {{\varOmega }}. $$

So, in terms of (2.1), this is equivalent to the fixed point equation

$$ P_{{\varOmega }}(2I-A)\widetilde{x}=\widetilde{x}. $$

This completes the proof. □

Taking $T\equiv I$, $G\equiv I$, $\mu =1$, and $\gamma =1$ in Theorem 3.2, we have the following corollary.

### Corollary 3.1

*Let* $\{x_{t}\}$ *be defined by*

$$ x_{t}=P_{C}\bigl[(I-\theta_{t}A){\varDelta}^{N}_{t}R_{t}x_{t}+ \theta_{t}\bigl(tVx _{t}+(1-t){\varDelta}^{N}_{t}R_{t}x_{t}\bigr)\bigr]. $$

*If* $\lim_{t\to 0}\theta_{t}=0$, *then* $x_{t}$ *converges strongly as* $t\to 0$ *to* $\widetilde{x}\in {{\varOmega }}:=\bigcap^{N}_{i=1} \operatorname {GMEP}( {\varTheta }_{i}, \varphi_{i}, B_{i})\cap \operatorname {GSVI}(C, B_{1}, B_{2})$, *which is the unique solution of the VI*

3.38 $$ \bigl\langle (A-I)\widetilde{x},\widetilde{x}-p\bigr\rangle \leq 0,\quad \forall p\in {{\varOmega }}. $$

### Proof

If $T\equiv I$, then $T_{r}$ in Lemma 2.8 is the identity mapping. Thus the result follows from Theorem 3.2. □

We are now in a position to prove the strong convergence of the sequence $\{x_{n}\}$ generated by the general explicit iterative scheme (3.3) to $\widetilde{x}\in {{\varOmega }}$, which is the unique solution to VI (3.2).

### Theorem 3.3

*Let* $\{x_{n}\}$ *be the sequence generated by the explicit algorithm* (3.3). *Let* $\{\alpha_{n}\}$, $\{\beta_{n}\}$, $\{r_{n} \}$, $\{\lambda_{n}\}$, $\{\nu_{n}\}$, *and* $\{r_{i, n}\}^{N}_{i=1}$ *satisfy the following conditions*:

1. $\{\alpha_{n}\}\subset [0, 1]$ *and* $\{\beta_{n}\}\subset (0, 1]$, $\alpha_{n}\to 0$ *and* $\beta_{n}\to 0$ *as* $n\to \infty $;
2. $\sum^{\infty }_{n=0}\beta_{n}=\infty $;
3. $\sum^{\infty }_{n=0}\vert \alpha_{n+1}-\alpha_{n}\vert <\infty $, *and* $\vert \beta_{n+1}-\beta_{n}\vert \leq o(\beta_{n+1})+\sigma_{n}$, $\sum^{\infty }_{n=0}\sigma_{n}<\infty $ (*the perturbed control condition*);
4. $\{r_{n}\}\subset (0,\infty )$, $\liminf_{n\to \infty }r_{n}>0$, *and* $\sum^{\infty }_{n=0}\vert r_{n+1}-r_{n}\vert <\infty $;
5. $\{\lambda_{n}\}\subset (0, \infty )$, $\lim_{n\to \infty }\lambda _{n}=\lambda >0$, *and* $\sum^{\infty }_{n=0}\vert \lambda_{n+1}-\lambda_{n}\vert < \infty $;
6. $\{\nu_{n}\}\subset (0,\infty )$, $\lim_{n\to \infty }\nu_{n}= \nu >0$, *and* $\sum^{\infty }_{n=0}\vert \nu_{n+1}-\nu_{n}\vert <\infty $;
7. $\{r_{i, n}\}\subset [c_{i}, d_{i}]\subset (0,2\mu_{i})$ $\forall i\in \{1, 2,\ldots, N\}$, *and* $\sum^{\infty }_{n=0}(\sum^{N}_{i=1}\vert r _{i,n+1}-r_{i,n}\vert )<\infty $.

*Then* $\{x_{n}\}$ *converges strongly to* $\widetilde{x}\in {{\varOmega }}:= \bigcap^{N}_{i=1}\operatorname {GMEP}({\varTheta }_{i}, \varphi_{i},B_{i}) \cap \operatorname {GSVI}(C,F_{1},F_{2})\cap \operatorname {Fix}(T)$, *which is the unique solution of VI* (3.2).

### Proof

First, note that from condition (C1), without loss of generality, we assume that $\alpha_{n}\tau <1$, $\beta_{n} \bar{\gamma }<1$ and $\frac{2\beta_{n}(\bar{\gamma }-1)}{1-\beta_{n}}<1$ for all $n\geq 0$. Let $\widetilde{x}\in {{\varOmega }}$ be the unique solution of VI (3.2). (The existence of *x̃* follows from Theorem 3.2.)

From now, we put $z_{n}=R_{n}x_{n}$, $u_{n}={\varDelta}^{N}_{n}z_{n}$, and $v_{n}=T_{r_{n}}u_{n}$. Take $p\in \varOmega $. Then $p=T_{r_{n}}p$ by Lemma 2.8(iii), $p={\varDelta}^{i}_{n}p$ ($=T^{({\varTheta }_{i}, \varphi_{i})}_{r_{i, n}}(I-r_{i, n}B_{i})p$) by Proposition 2.1(iii), and $p=R_{n}p$ by Lemma 1.1.

We divide the proof into several steps as follows.

*Step 1.* We show that $\{x_{n}\}$ is bounded. Indeed, utilizing Proposition 2.1(ii) and Proposition 2.2, we have

3.39 $$\begin{aligned} \Vert u_{n}-p \Vert &= \bigl\Vert T^{({\varTheta }_{N},\varphi_{N})}_{r_{N,n}}(I-r_{N,n}B _{N}){\varDelta}^{N-1}_{n}z_{n} -T^{({\varTheta }_{N},\varphi_{N})} _{r_{N,n}}(I-r_{N,n}B_{N}){\varDelta}^{N-1}_{n}p \bigr\Vert \\ &\leq \bigl\Vert (I-r_{N,n}B_{N}){\varDelta}^{N-1}_{n}z_{n}-(I-r_{N,n}B_{N}) {\varDelta}^{N-1}_{n}p \bigr\Vert \\ &\leq \bigl\Vert {{\varDelta}}^{N-1}_{n}z_{n}- {\varDelta}^{N-1}_{n}p \bigr\Vert \\ &\leq \cdots \\ &\leq \bigl\Vert {{\varDelta}}^{0}_{n}z_{n}- {\varDelta}^{0}_{n}p \bigr\Vert = \Vert z_{n}-p \Vert . \end{aligned}$$

It is easy from the nonexpansion of $R_{n}$ to see that

$$ \Vert z_{n}-p \Vert = \Vert R_{n}x_{n}-R_{n}p \Vert \leq \Vert x_{n}-p \Vert , $$

which together with the nonexpansion of $T_{r_{n}}$ and (3.39) implies that

3.40 $$ \Vert v_{n}-p \Vert = \Vert T_{r_{n}}u_{n}-T_{r_{n}}p \Vert \leq \Vert u_{n}-p \Vert \leq \Vert z_{n}-p \Vert \leq \Vert x_{n}-p \Vert . $$

From (3.3) and (3.40), we get

$$\begin{aligned} & \Vert x_{n+1}-p \Vert \\ &\quad \leq \bigl\Vert (I-\beta_{n}A)v_{n}+ \beta_{n}\bigl(\alpha_{n}\gamma Vx_{n}+(I- \alpha_{n}\mu G)v_{n}\bigr)-p \bigr\Vert \\ &\quad = \bigl\Vert (I-\beta_{n}A)v_{n}-(I- \beta_{n}A)p+\beta_{n}\bigl(\alpha_{n}\gamma Vx _{n}+(I-\alpha_{n}\mu G)v_{n}-p\bigr)+ \beta_{n}(I-A)p \bigr\Vert \\ &\quad \leq \bigl\Vert (I-\beta_{n}A)v_{n}-(I- \beta_{n}A)p \bigr\Vert +\beta_{n} \bigl\Vert \alpha_{n} \gamma Vx_{n}+(I-\alpha_{n}\mu G)v_{n}-p \bigr\Vert +\beta_{n} \bigl\Vert (I-A)p \bigr\Vert \\ &\quad = \bigl\Vert (I-\beta_{n}A)v_{n}-(I- \beta_{n}A)p \bigr\Vert \\ &\quad \quad{}+\beta_{n} \bigl\Vert (I-\alpha_{n}\mu G)v_{n}-(I-\alpha_{n}\mu G)p+\alpha_{n}( \gamma Vx_{n}-\mu Gp) \bigr\Vert +\beta_{n} \bigl\Vert (I-A)p \bigr\Vert \\ &\quad \leq (1-\beta_{n}\bar{\gamma }) \Vert v_{n}-p \Vert +\beta_{n}\bigl[ \bigl\Vert (I-\alpha_{n} \mu G)v_{n}-(I-\alpha_{n}\mu G)p \bigr\Vert \\ &\quad \quad{}+\alpha_{n}\bigl(\gamma \Vert Vx_{n}-Vp \Vert + \Vert \gamma Vp-\mu Gp \Vert \bigr)\bigr]+\beta_{n} \bigl\Vert (I-A)p \bigr\Vert \\ &\quad \leq (1-\beta_{n}\bar{\gamma }) \Vert x_{n}-p \Vert +\beta_{n}\bigl[(1-\alpha_{n} \tau ) \Vert x_{n}-p \Vert +\alpha_{n}\bigl(\gamma l \Vert x_{n}-p \Vert + \bigl\Vert (\gamma V-\mu G)p \bigr\Vert \bigr) \bigr] \\ &\quad \quad{}+\beta_{n} \Vert I-A \Vert \Vert p \Vert \\ &\quad =\bigl[1-\beta_{n}\bigl(\bar{\gamma }-1+\alpha_{n}( \tau -\gamma l)\bigr)\bigr] \Vert x_{n}-p \Vert + \beta_{n}\bigl[ \Vert I-A \Vert \Vert p \Vert + \alpha_{n} \bigl\Vert (\gamma V-\mu G)p \bigr\Vert \bigr] \\ &\quad \leq \bigl[1-\beta_{n}(\bar{\gamma }-1)\bigr] \Vert x_{n}-p \Vert +\beta_{n}\bigl[ \Vert I-A \Vert \Vert p \Vert + \bigl\Vert (\gamma V-\mu G)p \bigr\Vert \bigr] \\ &\quad =\bigl[1-\beta_{n}(\bar{\gamma }-1)\bigr] \Vert x_{n}-p \Vert +\beta_{n}(\bar{\gamma }-1) \frac{ \Vert I-A \Vert \Vert p \Vert + \Vert (\gamma V-\mu G)p \Vert }{\bar{\gamma }-1} \\ &\quad \leq \max \biggl\{ \Vert x_{n}-p \Vert , \frac{ \Vert I-A \Vert \Vert p \Vert + \Vert (\gamma V-\mu G)p \Vert }{\bar{ \gamma }-1}\biggr\} . \end{aligned}$$

By induction, we derive

$$ \Vert x_{n}-p \Vert \leq \max \biggl\{ \Vert x_{0}-p \Vert ,\frac{ \Vert I-A \Vert \Vert p \Vert + \Vert (\gamma V- \mu G)p \Vert }{\bar{\gamma }-1}\biggr\} ,\quad \forall n\geq 0. $$

This implies that $\{x_{n}\}$ is bounded and so are $\{Vx_{n}\}$, $\{u _{n}\}$, $\{v_{n}\}$, $\{w_{n}\}$, $\{z_{n}\}$, and $\{Gv_{n}\}$. As a consequence, with the control condition (C1), we get

3.41 $$ \Vert x_{n+1}-v_{n} \Vert \leq \beta_{n} \Vert w_{n}-Av_{n} \Vert \to 0\quad (n\to \infty ). $$

*Step 2.* We show that $\lim_{n\to \infty }\Vert x_{n+1}-x_{n}\Vert =0$. To this end, let $y_{n}=F_{2, \nu_{n}}x_{n}$, $y_{n-1}=F_{2, \nu_{n-1}}x _{n-1}$, $z_{n}=F_{1,\lambda_{n}}y_{n}$, and $z_{n-1}=F_{1, \lambda_{n-1}}y _{n-1}$. Then we derive

3.42 $$ \langle y-y_{n-1}, F_{2}y_{n-1}\rangle + \frac{1}{\nu_{n-1}}\langle y-y _{n-1}, y_{n-1}-x_{n-1} \rangle \geq 0,\quad \forall y\in C, $$

and

3.43 $$ \langle y-y_{n}, F_{2}y_{n}\rangle + \frac{1}{\nu_{n}}\langle y-y_{n},y _{n}-x_{n} \rangle \geq 0,\quad \forall y\in C. $$

Putting $y=y_{n}$ in (3.42) and $y=y_{n-1}$ in (3.43), we obtain

3.44 $$ \langle y_{n}-y_{n-1}, F_{2}y_{n-1} \rangle +\frac{1}{\nu_{n-1}}\langle y_{n}-y_{n-1}, y_{n-1}-x_{n-1}\rangle \geq 0 $$

and

3.45 $$ \langle y_{n-1}-y_{n}, F_{2}y_{n} \rangle +\frac{1}{\nu_{n}}\langle y _{n-1}-y_{n}, y_{n}-x_{n}\rangle \geq 0. $$

Adding up (3.44) and (3.45), we have

$$ \langle y_{n}-y_{n-1},F_{2}y_{n-1}-F_{2}y_{n} \rangle +\biggl\langle y_{n}-y _{n-1}, \frac{y_{n-1}-x_{n-1}}{\nu_{n-1}} - \frac{y_{n}-x_{n}}{\nu_{n}}\biggr\rangle \geq 0, $$

which together with the monotonicity of $F_{2}$ implies that

$$ \biggl\langle y_{n}-y_{n-1}, \frac{y_{n-1}-x_{n-1}}{\nu_{n-1}}- \frac{y_{n}-x _{n}}{\nu_{n}}\biggr\rangle \geq 0, $$

and hence

$$ \biggl\langle y_{n}-y_{n-1}, y_{n-1}-y_{n}+y_{n}-x_{n-1}- \frac{\nu_{n-1}}{ \nu_{n}}(y_{n}-x_{n})\biggr\rangle \geq 0. $$

It follows that

$$\begin{aligned} \Vert y_{n}-y_{n-1} \Vert ^{2} &\leq \biggl\langle y_{n}-y_{n-1},x_{n}-x_{n-1}+ \biggl(1-\frac{ \nu_{n-1}}{\nu_{n}}\biggr) (y_{n}-x_{n})\biggr\rangle \\ &\leq \Vert y_{n}-y_{n-1} \Vert \biggl( \Vert x_{n}-x_{n-1} \Vert +\frac{1}{\nu_{n}} \vert \nu_{n}- \nu_{n-1} \vert \Vert y_{n}-x_{n} \Vert \biggr), \end{aligned}$$

which immediately yields

3.46 $$ \Vert y_{n}-y_{n-1} \Vert \leq \Vert x_{n}-x_{n-1} \Vert +\frac{1}{\nu_{n}} \vert \nu_{n}- \nu_{n-1} \vert \Vert y_{n}-x_{n} \Vert . $$

By using arguments similar to those of (3.46), we get

3.47 $$ \Vert z_{n}-z_{n-1} \Vert \leq \Vert y_{n}-y_{n-1} \Vert +\frac{1}{\lambda_{n}} \vert \lambda _{n}-\lambda_{n-1} \vert \Vert z_{n}-y_{n} \Vert . $$

Substituting (3.46) for (3.47), we have

3.48 $$\begin{aligned} \Vert z_{n}-z_{n-1} \Vert &\leq \Vert y_{n}-y_{n-1} \Vert +\frac{1}{\lambda_{n}} \vert \lambda _{n}-\lambda_{n-1} \vert \Vert z_{n}-y_{n} \Vert \\ &\leq \Vert x_{n}-x_{n-1} \Vert +\frac{1}{\nu_{n}} \vert \nu_{n}-\nu_{n-1} \vert \Vert y_{n}-x _{n} \Vert +\frac{1}{\lambda_{n}} \vert \lambda_{n}- \lambda_{n-1} \vert \Vert z_{n}-y_{n} \Vert . \end{aligned}$$

Note that $v_{n}=T_{r_{n}}u_{n}$ and $v_{n-1}=T_{r_{n-1}}u_{n-1}$. By using arguments similar to those of (3.46), we obtain

3.49 $$ \Vert v_{n}-v_{n-1} \Vert \leq \Vert u_{n}-u_{n-1} \Vert +\frac{1}{r_{n}} \vert r_{n}-r_{n-1} \vert \Vert v_{n}-u_{n} \Vert . $$

Also, utilizing arguments similar to those of (3.25) in the proof of Theorem 3.1, we have

3.50 $$\begin{aligned} \Vert u_{n}-u_{n-1} \Vert &= \bigl\Vert {{\varDelta}}^{N}_{n}z_{n}-{\varDelta}^{N}_{n-1}z _{n-1} \bigr\Vert \\ &\leq \vert r_{N,n}-r_{N, n-1} \vert \biggl[ \bigl\Vert B_{N}{{\varDelta}}^{N-1}_{n}z_{n} \bigr\Vert +\frac{1}{r _{N,n}} \bigl\Vert T^{({\varTheta }_{N}, \varphi_{N})}_{r_{N, n}} (I-r_{N,n}B _{N}){\varDelta}^{N-1}_{n}z_{n} \\ &\quad{}-(I-r_{N, n}B_{N}){\varDelta}^{N-1}_{n}z_{n} \bigr\Vert \biggr]+\cdots + \vert r_{1, n}-r _{1, n-1} \vert \biggl[ \bigl\Vert B_{1}{{\varDelta}}^{0}_{n}z_{n} \bigr\Vert \\ &\quad{}+\frac{1}{r_{1, n}} \bigl\Vert T^{({\varTheta }_{1}, \varphi_{1})}_{r_{1, n}}(I-r _{1, n}B_{1}){\varDelta}^{0}_{n}z_{n}- (I-r_{1, n}B_{1}){\varDelta}^{0}_{n}z_{n} \bigr\Vert \biggr] \\ &\quad{}+ \bigl\Vert {{\varDelta}}^{0}_{n}z_{n}- {\varDelta}^{0}_{n-1}z _{n-1} \bigr\Vert \\ &\leq \widetilde{M}_{1}{ \sum^{N}_{i=1}} \vert r_{i, n}-r_{i, n-1} \vert + \Vert z_{n}-z_{n-1} \Vert , \end{aligned}$$

where $\widetilde{M}_{1}>0$ is a constant such that, for each $n\geq 0$,

$$ \sum^{N}_{i=1}\biggl[ \bigl\Vert B_{i}{{\varDelta}}^{i-1}_{n}z_{n} \bigr\Vert + \frac{1}{r_{i,n}} \bigl\Vert T^{({\varTheta }_{i},\varphi_{i})}_{r_{i,n}}(I-r _{i,n}B_{i}){\varDelta}^{i-1}_{n}z_{n} -(I-r_{i,n}B_{i}){\varDelta}^{i-1}_{n}z_{n} \bigr\Vert \biggr]\}\leq \widetilde{M}_{1}. $$

So it follows from (3.48), (3.49), and (3.50) that

3.51 $$\begin{aligned} \Vert v_{n}-v_{n-1} \Vert &\leq \Vert u_{n}-u_{n-1} \Vert +\frac{1}{r_{n}} \vert r_{n}-r_{n-1} \vert \Vert v_{n}-u_{n} \Vert \\ &\leq \widetilde{M}_{1}{ \sum^{N}_{i=1}} \vert r_{i,n}-r_{i,n-1} \vert + \Vert z_{n}-z_{n-1} \Vert +\frac{1}{r_{n}} \vert r _{n}-r_{n-1} \vert \Vert v_{n}-u_{n} \Vert \\ &\leq \widetilde{M}_{1}{ \sum^{N}_{i=1}} \vert r_{i,n}-r_{i,n-1} \vert + \Vert x_{n}-x_{n-1} \Vert +\frac{1}{\nu _{n}} \vert \nu_{n}-\nu_{n-1} \vert \Vert y_{n}-x_{n} \Vert \\ &\quad{}+\frac{1}{\lambda_{n}} \vert \lambda_{n}-\lambda_{n-1} \vert \Vert z_{n}-y_{n} \Vert +\frac{1}{r _{n}} \vert r_{n}-r_{n-1} \vert \Vert v_{n}-u_{n} \Vert . \end{aligned}$$

Since $\liminf_{n\to \infty }r_{n}>0$, $\lim_{n\to \infty }\lambda_{n}= \lambda >0$, and $\lim_{n\to \infty }\nu_{n}=\nu >0$, it is easy to see from (3.51) that, for each $n\geq 0$,

3.52 $$ \begin{aligned}[b] \Vert v_{n}-v_{n-1} \Vert \leq {}&\Vert x_{n}-x_{n-1} \Vert +\widetilde{M}\Biggl[{ \sum ^{N}_{i=1}} \vert r_{i,n}-r_{i,n-1} \vert + \vert \nu_{n}-\nu_{n-1} \vert \\ &{} + \vert \lambda_{n}- \lambda_{n-1} \vert + \vert r_{n}-r_{n-1} \vert \Biggr],\end{aligned} $$

where $\widetilde{M}>0$ is a constant such that

$$ \sup_{n\geq 0}\biggl\{ \widetilde{M}_{1}+ \frac{1}{\nu_{n}} \Vert y_{n}-x_{n} \Vert + \frac{1}{ \lambda_{n}} \Vert z_{n}-y_{n} \Vert + \frac{1}{r_{n}} \Vert v_{n}-u_{n} \Vert \biggr\} \leq \widetilde{M}. $$

Now, simple calculations yield that

$$\begin{aligned} w_{n}-w_{n-1}&=\alpha_{n}\gamma Vx_{n}+(I-\alpha_{n}\mu G)v_{n}- \alpha_{n-1}\gamma Vx_{n-1}-(I-\alpha_{n-1}\mu G)v_{n-1} \\ &=(\alpha_{n}-\alpha_{n-1}) (\gamma Vx_{n-1}-\mu Gv_{n-1})+\alpha_{n} \gamma (Vx_{n}-Vx_{n-1}) \\ &\quad{}+(I-\alpha_{n}\mu G)v_{n}-(I-\alpha_{n} \mu G)v_{n-1}. \end{aligned}$$

In terms of (3.52) and Lemma 2.6, we obtain

3.53 $$\begin{aligned} \Vert w_{n}-w_{n-1} \Vert &\leq \vert \alpha_{n}-\alpha_{n-1} \vert \bigl(\gamma \Vert Vx_{n-1} \Vert + \mu \Vert Gv_{n-1} \Vert \bigr)+ \alpha_{n}\gamma l \Vert x_{n}-x_{n-1} \Vert \\ &\quad{}+(1-\tau \alpha_{n}) \Vert v_{n}-v_{n-1} \Vert \\ &\leq \vert \alpha_{n}-\alpha_{n-1} \vert \bigl(\gamma \Vert Vx_{n-1} \Vert +\mu \Vert Gv_{n-1} \Vert \bigr)+ \alpha_{n}\gamma l \Vert x_{n}-x_{n-1} \Vert \\ &\quad{}+(1-\tau \alpha_{n}) \Vert x_{n}-x_{n-1} \Vert +\widetilde{M}\Biggl[{ \sum^{N}_{i=1}} \vert r_{i,n}-r_{i,n-1} \vert \\ &\quad{}+ \vert \nu_{n}-\nu_{n-1} \vert + \vert \lambda_{n}-\lambda_{n-1} \vert + \vert r_{n}-r_{n-1} \vert \Biggr] \\ &= \vert \alpha_{n}-\alpha_{n-1} \vert \bigl(\gamma \Vert Vx_{n-1} \Vert +\mu \Vert Gv_{n-1} \Vert \bigr)+ \bigl(1- \alpha_{n}(\tau -\gamma l)\bigr) \Vert x_{n}-x_{n-1} \Vert \\ &\quad{}+\widetilde{M}\Biggl[{ \sum^{N}_{i=1}} \vert r_{i,n}-r_{i,n-1} \vert + \vert \nu_{n}- \nu_{n-1} \vert + \vert \lambda_{n}- \lambda_{n-1} \vert + \vert r_{n}-r_{n-1} \vert \Biggr] \\ &\leq \Vert x_{n}-x_{n-1} \Vert +\widetilde{M}_{2} \Biggl[{ \sum^{N}_{i=1}} \vert r_{i,n}-r_{i,n-1} \vert + \vert \alpha_{n}- \alpha_{n-1} \vert \\ &\quad{}+ \vert \nu_{n}-\nu_{n-1} \vert + \vert \lambda_{n}-\lambda_{n-1} \vert + \vert r_{n}-r_{n-1} \vert \Biggr], \end{aligned}$$

where $\widetilde{M}_{2}=\sup_{n\geq 0}\{\gamma \Vert Vx_{n}\Vert +\mu \Vert Gv _{n}\Vert +\widetilde{M}\}$. By (3.53) and Lemma 2.5, we derive

3.54 $$\begin{aligned} \Vert x_{n+1}-x_{n} \Vert &\leq \bigl\Vert (I- \beta_{n}A)v_{n}+\beta_{n}w_{n}-(I- \beta _{n-1}A)v_{n-1}-\beta_{n-1}w_{n-1} \bigr\Vert \\ &\leq \bigl\Vert (I-\beta_{n}A) (v_{n}-v_{n-1}) \bigr\Vert + \vert \beta_{n}-\beta_{n-1} \vert \Vert A \Vert \Vert v_{n-1} \Vert \\ &\quad{}+\beta_{n} \Vert w_{n}-w_{n-1} \Vert + \vert \beta_{n}-\beta_{n-1} \vert \Vert w_{n-1} \Vert \\ &\leq (1-\beta_{n}\bar{\gamma }) \Vert v_{n}-v_{n-1} \Vert +\beta_{n}\Biggl[ \Vert x_{n}-x _{n-1} \Vert +\widetilde{M}_{2}\Biggl({ \sum^{N}_{i=1}} \vert r_{i,n}-r_{i,n-1} \vert \\ &\quad{}+ \vert \alpha_{n}-\alpha_{n-1} \vert + \vert \nu_{n}-\nu_{n-1} \vert + \vert \lambda_{n}- \lambda _{n-1} \vert + \vert r_{n}-r_{n-1} \vert \Biggr)\Biggr] + \vert \beta_{n}-\beta_{n-1} \vert \widetilde{M}_{3} \\ &\leq (1-\beta_{n}\bar{\gamma })\Biggl[ \Vert x_{n}-x_{n-1} \Vert +\widetilde{M}\Biggl( { \sum^{N}_{i=1}} \vert r_{i,n}-r_{i,n-1} \vert + \vert \nu_{n}- \nu_{n-1} \vert + \vert \lambda_{n}- \lambda_{n-1} \vert \\ &\quad{}+ \vert r_{n}-r_{n-1} \vert \Biggr)\Biggr]+ \beta_{n}\Biggl[ \Vert x_{n}-x_{n-1} \Vert + \widetilde{M}_{2}\Biggl( { \sum^{N}_{i=1}} \vert r_{i,n}-r_{i,n-1} \vert + \vert \alpha_{n}-\alpha_{n-1} \vert \\ &\quad{}+ \vert \nu_{n}-\nu_{n-1} \vert + \vert \lambda_{n}-\lambda_{n-1} \vert + \vert r_{n}-r_{n-1} \vert \Biggr)\Biggr]+ \vert \beta_{n}-\beta_{n-1} \vert \widetilde{M}_{3} \\ &\leq \bigl(1-\beta_{n}(\bar{\gamma }-1)\bigr) \Vert x_{n}-x_{n-1} \Vert +\bigl(1-\beta_{n}(\bar{ \gamma }-1)\bigr)\widetilde{M}_{2}\Biggl({ \sum ^{N}_{i=1}} \vert r_{i,n}-r_{i,n-1} \vert \\ &\quad{}+ \vert \alpha_{n}-\alpha_{n-1} \vert + \vert \nu_{n}-\nu_{n-1} \vert + \vert \lambda_{n}- \lambda _{n-1} \vert + \vert r_{n}-r_{n-1} \vert \Biggr) + \vert \beta_{n}-\beta_{n-1} \vert \widetilde{M}_{3} \\ &\leq \bigl(1-\beta_{n}(\bar{\gamma }-1)\bigr) \Vert x_{n}-x_{n-1} \Vert +\widetilde{M} _{2}\Biggl({ \sum^{N}_{i=1}} \vert r_{i,n}-r_{i,n-1} \vert + \vert \alpha_{n}-\alpha_{n-1} \vert \\ &\quad{}+ \vert \nu_{n}-\nu_{n-1} \vert + \vert \lambda_{n}-\lambda_{n-1} \vert + \vert r_{n}-r_{n-1} \vert \Biggr)+ \vert \beta_{n}- \beta_{n-1} \vert \widetilde{M}_{3} \\ &\leq \bigl(1-\beta_{n}(\bar{\gamma }-1)\bigr) \Vert x_{n}-x_{n-1} \Vert +\widetilde{M} _{2}\Biggl({ \sum^{N}_{i=1}} \vert r_{i,n}-r_{i,n-1} \vert + \vert \alpha_{n}-\alpha_{n-1} \vert \\ &\quad{}+ \vert \nu_{n}-\nu_{n-1} \vert + \vert \lambda_{n}-\lambda_{n-1} \vert + \vert r_{n}-r_{n-1} \vert \Biggr)+\bigl(o( \beta_{n})+ \sigma_{n-1}\bigr)\widetilde{M}_{3}, \end{aligned}$$

where $\widetilde{M}_{3}=\sup_{n\geq 0}\{\Vert A\Vert \Vert v_{n}\Vert +\Vert w_{n}\Vert \}$. By taking $s_{n+1}=\Vert x_{n+1}-x_{n}\Vert $, $\omega_{n}=\beta_{n}(\bar{ \gamma }-1)$, $\omega_{n}\delta_{n}=\widetilde{M}_{3}o(\beta_{n})$, and

$$ \gamma_{n}=\sigma_{n-1}\widetilde{M}_{3}+ \widetilde{M}_{2}\Biggl({ \sum^{N}_{i=1}} \vert r_{i,n}-r_{i,n-1} \vert + \vert \alpha_{n}-\alpha_{n-1} \vert + \vert \nu _{n}-\nu_{n-1} \vert + \vert \lambda_{n}- \lambda_{n-1} \vert + \vert r_{n}-r_{n-1} \vert \Biggr), $$

we deduce from (3.54) that

$$ s_{n+1}\leq (1-\omega_{n})s_{n}+ \omega_{n}\delta_{n}+\gamma_{n}. $$

Hence, by conditions (C2)–(C7) and Lemma 2.2, we obtain

$$ \lim_{n\to \infty } \Vert x_{n+1}-x_{n} \Vert =0. $$

*Step 3.* We show that $\lim_{n\to \infty }\Vert x_{n+1}-w_{n}\Vert =0$. Indeed, from (3.41) and condition (C1), we derive

$$\begin{aligned} \Vert x_{n+1}-w_{n} \Vert &\leq \Vert x_{n+1}-v_{n} \Vert + \Vert v_{n}-w_{n} \Vert \\ &\leq \beta_{n} \Vert w_{n}-Av_{n} \Vert + \alpha_{n} \Vert \gamma Vx_{n}-\mu Gv_{n} \Vert \to 0\quad (n\to \infty ). \end{aligned}$$

*Step 4.* We show that $\lim_{n\to \infty }\Vert x_{n}-w_{n}\Vert =0$. In fact, by Step 2 and Step 3, we get

$$ \Vert x_{n}-w_{n} \Vert \leq \Vert x_{n}-x_{n+1} \Vert + \Vert x_{n+1}-w_{n} \Vert \to 0\quad (n \to \infty ). $$

*Step 5.* We show that $\lim_{n\to \infty }\Vert x_{n}-z_{n}\Vert =0$ and $\lim_{n\to \infty }\Vert x_{n}-Rx_{n}\Vert =0$. In fact, we first derive $\lim_{n\to \infty }\Vert x_{n}-z_{n}\Vert =0$ by using arguments similar to those of (3.9) in the proof of Theorem 3.1, and then we obtain $\lim_{n\to \infty }\Vert x_{n}-Rx_{n}\Vert =0$ by using arguments similar to those of (3.37) in the proof of Theorem 3.2.

*Step 6.* We show that $\lim_{n\to \infty }\Vert z_{n}-u_{n}\Vert =0$ and $\lim_{n\to \infty }\Vert x_{n}-{\varDelta}^{N}_{n}x_{n}\Vert =0$. In fact, by using arguments similar to those of (3.12) and (3.13) in the proof of Theorem 3.1, we obtain the desired conclusions.

*Step 7.* We show that $\lim_{n\to \infty }\Vert u_{n}-v_{n}\Vert =0$ and $\lim_{n\to \infty }\Vert x_{n}-T_{r_{n}}x_{n}\Vert =0$. In fact, by using arguments similar to those of (3.14) and (3.15) in the proof of Theorem 3.1, we obtain the desired conclusions.

*Step 8.* We show that $\limsup_{n\to \infty }\langle (I-A) \widetilde{x},x_{n}-\widetilde{x}\rangle \leq 0$. To this end, take a subsequence $\{x_{n_{k}}\}$ of $\{x_{n}\}$ such that

$$ \limsup_{n\to \infty }\bigl\langle (I-A)\widetilde{x},x_{n}- \widetilde{x} \bigr\rangle =\lim_{k\to \infty }\bigl\langle (I-A) \widetilde{x}, x_{n_{k}}- \widetilde{x}\bigr\rangle . $$

Without loss of generality, we may assume that $x_{n_{k}}\rightharpoonup \hat{x}$. Utilizing Steps 5, 6, and 7 and arguments similar to those of Steps 2, 3, and 4 in the proof of Theorem 3.2, we derive $\hat{x} \in {{\varOmega }}$. Thus, from VI (3.2), we conclude

$$ \limsup_{n\to \infty }\bigl\langle (I-A)\widetilde{x}, x_{n}-\widetilde{x} \bigr\rangle =\lim_{k\to \infty }\bigl\langle (I-A)\widetilde{x}, x_{n_{k}}- \widetilde{x}\bigr\rangle =\bigl\langle (I-A)\widetilde{x},\hat{x}- \widetilde{x}\bigr\rangle \leq 0. $$

*Step 9.* We show that $\lim_{n\to \infty }\Vert x_{n}-\widetilde{x} \Vert =0$. Note that $\widetilde{x}\in {{\varOmega }}$. From (3.3), $\widetilde{x}=R_{n}\widetilde{x}$, $\widetilde{x}={\varDelta}^{N} _{n}\widetilde{x}$, and $\widetilde{x}=T_{r_{n}}\widetilde{x}$, we obtain

$$ w_{n}-\widetilde{x}=(I-\alpha_{n}\mu G)v_{n}-(I- \alpha_{n}\mu G) \widetilde{x}+\alpha_{n}(\gamma Vx_{n}-\mu G\widetilde{x}) $$

and

$$\begin{aligned} x_{n+1}-\widetilde{x}&=x_{n+1}-(I-\beta_{n}A)v_{n}- \beta_{n}w_{n}+(I- \beta_{n}A)v_{n}+ \beta_{n}w_{n}-\widetilde{x} \\ &=x_{n+1}-(I-\beta_{n}A)v_{n}- \beta_{n}w_{n}+(I-\beta_{n}A) (v_{n}- \widetilde{x}) +\beta_{n}(w_{n}-\widetilde{x})+ \beta_{n}(I-A) \widetilde{x}. \end{aligned}$$

Applying (2.1), (3.40) and Lemmas 2.1, 2.5, and 2.6, we deduce that

$$\begin{aligned} \Vert w_{n}-\widetilde{x} \Vert ^{2}&= \bigl\Vert (I- \alpha_{n}\mu G)v_{n}-(I-\alpha_{n} \mu G) \widetilde{x}+\alpha_{n}(\gamma Vx_{n}-\mu G\widetilde{x}) \bigr\Vert ^{2} \\ &\leq \bigl\Vert (I-\alpha_{n}\mu G)v_{n}-(I- \alpha_{n}\mu G)\widetilde{x} \bigr\Vert ^{2}+2 \alpha_{n}\langle \gamma Vx_{n}-\mu G\widetilde{x}, w_{n}- \widetilde{x}\rangle \\ &\leq (1-\alpha_{n}\tau )^{2} \Vert v_{n}- \widetilde{x} \Vert ^{2}+2\alpha_{n} \Vert \gamma Vx_{n}-\mu G\widetilde{x} \Vert \Vert w_{n}- \widetilde{x} \Vert \\ &\leq \Vert x_{n}-\widetilde{x} \Vert ^{2}+2 \alpha_{n} \Vert \gamma Vx_{n}-\mu G \widetilde{x} \Vert \Vert w_{n}-\widetilde{x} \Vert , \end{aligned}$$

and hence

3.55 $$\begin{aligned} & \Vert x_{n+1}-\widetilde{x} \Vert ^{2} \\ &\quad = \bigl\Vert (I-\beta_{n}A) (v_{n}- \widetilde{x})+\beta_{n}(w_{n}-\widetilde{x})+ \beta_{n}(I-A)\widetilde{x}+x_{n+1}-(I-\beta_{n}A)v_{n}- \beta_{n}w _{n} \bigr\Vert ^{2} \\ &\quad \leq \bigl\Vert (I-\beta_{n}A) (v_{n}- \widetilde{x}) \bigr\Vert ^{2}+2\beta_{n}\langle w _{n}-\widetilde{x},x_{n+1}-\widetilde{x}\rangle \\ &\quad \quad{}+2\beta_{n}\bigl\langle (I-A)\widetilde{x},x_{n+1}- \widetilde{x}\bigr\rangle +2 \bigl\langle x_{n+1}-(I- \beta_{n}A)v_{n}-\beta_{n}w_{n},x_{n+1}- \widetilde{x}\bigr\rangle \\ &\quad \leq \bigl\Vert (I-\beta_{n}A) (v_{n}- \widetilde{x}) \bigr\Vert ^{2}+2\beta_{n}\langle w _{n}-\widetilde{x},x_{n+1}-\widetilde{x}\rangle +2 \beta_{n}\bigl\langle (I-A) \widetilde{x},x_{n+1}- \widetilde{x}\bigr\rangle \\ &\quad \leq (1-\beta_{n}\bar{\gamma })^{2} \Vert v_{n}-\widetilde{x} \Vert ^{2}+2\beta _{n} \Vert w_{n}-\widetilde{x} \Vert \Vert x_{n+1}- \widetilde{x} \Vert +2\beta_{n} \bigl\langle (I-A) \widetilde{x},x_{n+1}-\widetilde{x}\bigr\rangle \\ &\quad \leq (1-\beta_{n}\bar{\gamma })^{2} \Vert x_{n}-\widetilde{x} \Vert ^{2}+\beta _{n}\bigl( \Vert w_{n}-\widetilde{x} \Vert ^{2}+ \Vert x_{n+1}-\widetilde{x} \Vert ^{2}\bigr) +2 \beta_{n}\bigl\langle (I-A)\widetilde{x},x_{n+1}-\widetilde{x} \bigr\rangle \\ &\quad \leq (1-\beta_{n}\bar{\gamma })^{2} \Vert x_{n}-\widetilde{x} \Vert ^{2}+\beta _{n}\bigl[ \Vert x_{n}-\widetilde{x} \Vert ^{2}+2 \alpha_{n} \Vert \gamma Vx_{n}-\mu G \widetilde{x} \Vert \Vert w_{n}-\widetilde{x} \Vert \bigr] \\ &\quad \quad{}+\beta_{n} \Vert x_{n+1}-\widetilde{x} \Vert ^{2}+2\beta_{n}\bigl\langle (I-A) \widetilde{x},x_{n+1}-\widetilde{x}\bigr\rangle \\ &\quad =\bigl[(1-\beta_{n}\bar{\gamma })^{2}+ \beta_{n}\bigr] \Vert x_{n}-\widetilde{x} \Vert ^{2}+2\alpha_{n}\beta_{n} \Vert \gamma Vx_{n}-\mu G\widetilde{x} \Vert \Vert w_{n}- \widetilde{x} \Vert +\beta_{n} \Vert x_{n+1}-\widetilde{x} \Vert ^{2} \\ &\quad \quad{}+2\beta_{n}\bigl\langle (I-A)\widetilde{x},x_{n+1}- \widetilde{x}\bigr\rangle . \end{aligned}$$

It then follows from (3.55) that

$$\begin{aligned} & \Vert x_{n+1}-\widetilde{x} \Vert ^{2} \\ &\quad \leq \frac{(1-\beta_{n}\bar{\gamma })^{2}+\beta_{n}}{1-\beta_{n}} \Vert x _{n}-\widetilde{x} \Vert ^{2}+\frac{\beta_{n}}{1-\beta_{n}}\bigl[2\alpha_{n} \Vert \gamma Vx_{n}-\mu G\widetilde{x} \Vert \Vert w_{n}- \widetilde{x} \Vert \\ &\quad \quad{}+2\bigl\langle (I-A) \widetilde{x},x_{n+1}- \widetilde{x}\bigr\rangle \bigr] \\ &\quad =\biggl(1-\frac{2\beta_{n}(\bar{\gamma }-1)}{1-\beta_{n}}\biggr) \Vert x_{n}- \widetilde{x} \Vert ^{2}+\frac{2\beta_{n}(\bar{\gamma }-1)}{1-\beta_{n}} \cdot \frac{1}{2(\bar{\gamma }-1)} \bigl[2\alpha_{n} \Vert \gamma Vx_{n}-\mu G \widetilde{x} \Vert \Vert w_{n}-\widetilde{x} \Vert \\ &\quad \quad{}+\beta_{n}\bar{\gamma }^{2} \Vert x_{n}-\widetilde{x} \Vert ^{2}+2\bigl\langle (I-A) \widetilde{x},x_{n+1}-\widetilde{x}\bigr\rangle \bigr] \\ &\quad =(1-\xi_{n}) \Vert x_{n}-\widetilde{x} \Vert ^{2}+\xi_{n}\delta_{n}, \end{aligned}$$

where $\xi_{n}=\frac{2\beta_{n}(\bar{\gamma }-1)}{1-\beta_{n}}$, $\delta_{n}=\frac{1}{2(\bar{\gamma }-1)}[2\alpha_{n}\Vert \gamma Vx_{n}- \mu G\widetilde{x}\Vert \Vert w_{n}-\widetilde{x}\Vert +\beta_{n}\bar{\gamma } ^{2}\Vert x_{n}-\widetilde{x}\Vert ^{2}+2\langle (I-A)\widetilde{x},x_{n+1}- \widetilde{x}\rangle ]$. It can be readily seen from Step 2 and conditions (C1) and (C2) that $\xi_{n}\to 0$, $\sum^{\infty }_{n=0}\xi _{n}=\infty $, and $\limsup_{n\to \infty }\delta_{n}\leq 0$. By Lemma 2.2, we conclude that $\lim_{n\to \infty }\Vert x_{n}- \widetilde{x}\Vert =0$. This completes the proof. □

Taking $T\equiv I$, $G\equiv I$, $\mu =1$, and $\gamma =1$ in Theorem 3.3, we have the following corollary.

### Corollary 3.2

*Let* $\{x_{n}\}$ *be generated by the following iterative algorithm*:

$$ \textstyle\begin{cases} w_{n}=\alpha_{n} Vx_{n}+(1-\alpha_{n}){\varDelta}^{N}_{n}R_{n}x_{n}, \\ x_{n+1}=P_{C}[(I-\beta_{n}A){\varDelta}^{N}_{n}R_{n}x_{n}+\beta_{n}w _{n}],\quad \forall n\geq 1. \end{cases} $$

*Assume that the sequences* $\{\alpha_{n}\}$, $\{\beta_{n}\}$, $\{\lambda _{n}\}$, $\{\nu_{n}\}$, *and* $\{r_{i, n}\}^{N}_{i=1}$ *satisfy conditions* (C1)*–*(C3) *and* (C5)*–*(C7) *in Theorem *3.3. *Then* $\{x_{n}\}$ *converges strongly to* $\widetilde{x}\in {{\varOmega }}:= \bigcap^{N}_{i=1}\operatorname {GMEP}( {\varTheta }_{i}, \varphi_{i}, B_{i})\cap \operatorname {GSVI}(C, F_{1}, F_{2})$, *which is the unique solution of VI* (3.38).

### Remark 3.1

Compared with Proposition 3.3, Theorem 3.4, and Theorem 3.7 in [11], respectively, our Theorems 3.1, 3.2, and 3.3 improve and develop them in the following aspects:

(i) GSVI (1.3) with solutions being also fixed points of a continuous pseudocontinuous mapping in [12, Proposition 3.3, Theorem 3.4, and Theorem 3.7] is extended to GSVI (1.3) with solutions being also common solutions of a finite family of generalized mixed equilibrium problems (GMEPs) and fixed points of a continuous pseudocontinuous mapping in our Theorems 3.1, 3.2, and 3.3;
(ii) in the argument process of our Theorems 3.1, 3.2, and 3.3, we use the variable parameters $\lambda_{t}$ and $\nu_{t}$ (resp., $\lambda_{n}$ and $\nu_{n}$) in place of the fixed parameters *λ* and *ν* in the proof of [12, Proposition 3.3, Theorem 3.4, and Theorem 3.7], and additionally deal with a pool of variable parameters $\{r_{i, t}\}^{N}_{i=1}$ (resp., $\{r_{i, n}\}^{N}_{i=1}$) involving a finite family of GMEPs;
(iii) the iterative schemes in our Theorems 3.1, 3.2, and 3.3 are more advantageous and more flexible than the iterative schemes in [12, Proposition 3.3, Theorem 3.4, and Theorem 3.7], because they can be applied to solving three problems (i.e., GSVI (1.3), a finite family of GMEPs, and the fixed point problem of a continuous pseudocontractive mapping) and involve much more parameter sequences;
(iv) it is worth emphasizing that our general implicit iterative scheme (3.1) is very different from Jung’s composite implicit iterative scheme in [12], because the term “$T_{r_{t}}Rx_{t}$” in Jung’s implicit scheme is replaced by the term “$T_{r_{t}}{{\varDelta}}^{N}_{t}R _{t} x_{t}$” in our implicit scheme (3.1). Moreover, the term “$T _{r_{n}}Rx_{n}$” in Jung’s explicit scheme is replaced by the term “$T_{r_{n}}{{\varDelta}}^{N}_{n}R_{n}x_{n}$” in our explicit scheme (3.3).

## Numerical examples

The purpose of this section is to give two examples and numerical results to illustrate the applicability, effectiveness, and stability of our algorithm.

### Example 4.1

(Example of Theorem 3.3)

Let $H=\mathbf{R}$ and $C=[0, 100]$. Let the inner product $\langle \cdot , \cdot \rangle : \mathbf{R}\times \mathbf{R}\rightarrow {\mathbf{R}}$ be defined by $\langle x, y \rangle =xy$. Let $N=2$, $Vx=2x$, $Gx=\frac{1}{2}x$, $Tx=x$, $B_{1}x= \frac{1}{2}x$, $B_{2}x=\frac{1}{3}x$, $F_{1}x=\frac{1}{2}x$, $F_{2}x=x$, $\varTheta_{1}(x, y)=y^{2}-x^{2}$, $\varTheta_{2}(x, y)=-3x^{2}+xy+2y^{2}$, $\varphi_{1}x=x^{2}$, $\varphi_{2}x=0$, and $Ax=\frac{3}{2}x$. Let $\alpha_{n}=\frac{1}{n}$, $\beta_{n}=\frac{1}{3(n+1)}$, $r_{n}=1$, $r_{1,n}= \frac{1}{2}$, $r_{2,n}=1$, $\lambda_{n}=1$, $\nu_{n}=\frac{1}{2}$, $\gamma = \frac{1}{8}$, $\mu =\frac{2}{3}$. It is easy to calculate that $T^{({\varTheta }_{1}, \varphi_{1})}_{r_{1, n}}x=\frac{1}{3}x$, $T^{( {\varTheta }_{2}, \varphi_{2})}_{r_{2, n}}x=\frac{1}{6}x$, $T_{r_{n}}x=x$, $F_{1, \lambda_{n}}x=\frac{1}{2}x$, and $F_{2, \nu_{n}}x=\frac{1}{2}x$. Choose an arbitrary initial guess $x_{1}=4$. We get the numerical results of Algorithm (3.3).

Table 1 shows the value of the sequence $\{x_{n}\}$.

**Table 1:** The values of $x_{n}$

*n*	$x_{n}$
1	4.0000
2	1.8261 × 10^−1^
3	3.3191 × 10^−3^
4	3.7633 × 10^−5^
5	3.2426 × 10^−7^
6	2.3546 × 10^−9^
7	1.5285 × 10^−11^
8	9.1892 × 10^−14^
9	5.2325 × 10^−16^
10	2.8636 × 10^−18^
11	1.5212 × 10^−20^
12	7.8994 × 10^−23^

**Figure 1:**
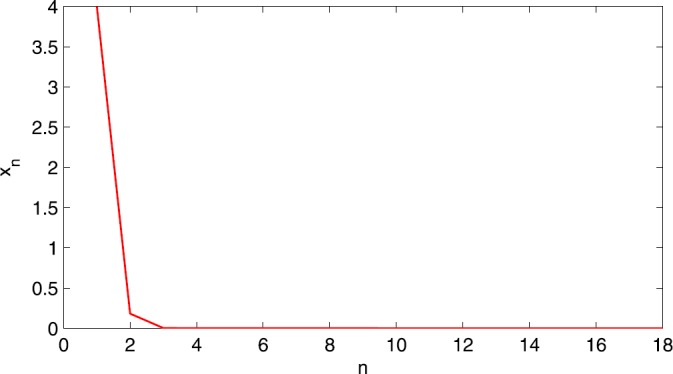
The convergence of $\{x_{n}\}$ with initial $x_{1}=4$

Figure 1 shows the convergence of the iterative sequence of Algorithm (3.3).

Solution: We can see from both Table 1 and Fig. 1 that the sequence $\{x_{n}\}$ converges to 0, that is, 0 is the solution in Example 4.1. In addition, it is also easy to check from Example 4.1 that $\bigcap^{2}_{i=1}\operatorname {GMEP}({\varTheta }_{i}, \varphi_{i}, B_{i}) \cap \operatorname {GSVI}(C, F_{1}, F_{2})\cap \operatorname {Fix}(T)=\{0\}$. Therefore, the iterative algorithm of Theorem 3.3 is efficient.

### Example 4.2

(Example of Theorem 3.7 in [12])

Let $H=\mathbf{R}$ and $C=[0, 100]$. Let the inner product $\langle \cdot , \cdot \rangle : \mathbf{R}\times \mathbf{R}\rightarrow {\mathbf{R}}$ be defined by $\langle x, y\rangle =xy$. Let $Vx=2x$, $Gx=\frac{1}{2}x$, $Tx=x$, $F_{1}x=\frac{1}{2}x$, $F_{2}x=x$, and $Ax=\frac{3}{2}x$. Let $\alpha_{n}=\frac{1}{n}$, $\beta _{n}=\frac{1}{3(n+1)}$, $r_{n}=1$, $\lambda =1$, $\nu =\frac{1}{2}$, $\gamma =\frac{1}{8}$, $\mu =\frac{2}{3}$. Choose an arbitrary initial guess $x_{1}=4$. We get the numerical results of Algorithm (1.5) (Algorithm (3.10) of [12]).

Table 2 shows the value of the sequence $\{x_{n}\}$.

**Table 2:** The values of $x_{n}$

*n*	$x_{n}$
1	4.0000
2	1.0278
3	2.5219 × 10^−1^
4	6.1587 × 10^−2^
5	1.5055 × 10^−2^
6	3.6870 × 10^−3^
7	9.0468 × 10^−4^
8	2.2236 × 10^−4^
9	5.4731 × 10^−5^
10	1.3488 × 10^−5^
11	3.3278 × 10^−6^
12	8.2181 × 10^−7^

**Figure 2:**
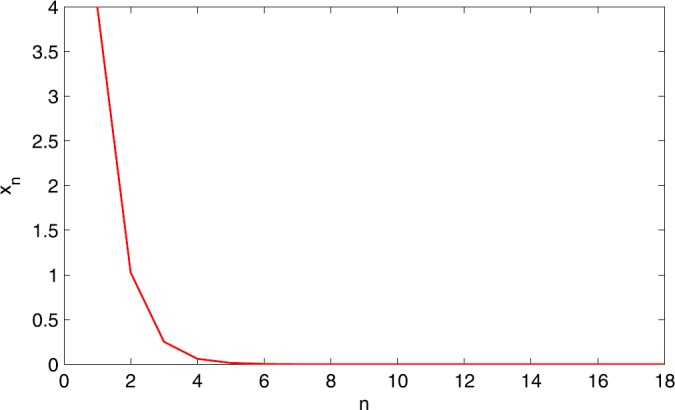
The convergence of $\{x_{n}\}$ with initial $x_{1}=4$

The Fig. 2 shows the convergence of the iterative sequence of Algorithm (1.5).

Solution: We can see from both Table 2 and Fig. 2 that the sequence $\{x_{n}\}$ converges to 0, that is, 0 is the solution in Example 4.2. In addition, it is also easy to check from Example 4.2 that $\operatorname {GSVI}(C, F_{1}, F_{2})\cap \operatorname {Fix}(T)=\{0\}$.

### Remark 4.1

From Tables 1, 2 and Figs. 1, 2, it is readily seen that the convergence of $\{x_{n}\}$ to 0 in Example 4.1 is faster than the one of $\{x_{n}\}$ to 0 in Example 4.2. Therefore, our algorithm is more applicable, efficient, and stable than the algorithm in [12].

## Application

In this section, applying our main result Theorem 3.3, we can prove strong convergence theorems for approximating the solution of the standard constrained convex optimization problem.

Let *C* be a closed convex subset of *H*. The standard constrained convex optimization problem is to find $x^{\ast }\in C$ such that

5.1 $$ f\bigl(x^{\ast }\bigr)=\min_{x\in C}f(x), $$

where $f : C\to {\mathbf{R}}$ is a convex, Fréchet differentiable function. The set of the solutions of (5.1) is denoted by $\varPhi_{f}$.

### Lemma 5.1

(Optimality condition, [25])

*A necessary condition of optimality for a point* $x^{\ast }\in C$ *to be a solution of the minimization problem* (5.1) *is that* $x^{\ast }$ *solves the variational inequality*

5.2 $$ \bigl\langle \nabla f\bigl(x^{\ast }\bigr), x-x^{\ast }\bigr\rangle \geq 0 $$

*for all* $x\in C$. *Equivalently*, $x^{\ast }\in C$ *solves the fixed point equation*

$$ x^{\ast }=P_{C}(I-\lambda \nabla f)x^{\ast } $$

*for every* $\lambda >0$. *If*, *in addition*, *f* *is convex*, *then the optimality condition* (5.2) *is also sufficient*.

### Theorem 5.1

*Let* *C* *be a nonempty closed convex subset of a real Hilbert space H*. *Let* $f_{i}$ ($i=1,2,\ldots, N$): $C\to {\mathbf{R}}$ *be a real*-*valued convex function with the gradient* $\nabla f_{i}$ *being* $\frac{1}{L_{f_{i}}}$-*inverse strongly monotone and continuous with* $L_{f_{i}}>0$. *Let* ${\varTheta }_{i}$, $\varphi_{i}$, *A*, *V*, *G*, $F_{1}$, $F _{2}$, $R_{n}$, $F_{1, \lambda_{n}}$, $F_{2, \nu_{n}}$, $T_{r_{n}}$, *and* $T^{({\varTheta }_{i},\varphi_{i})}_{r_{i,n}}$ *be defined as in Theorem *3.3. *Given* $x_{1}\in C$ *and let* $\{x_{n}\}$ *be the sequence generated by the following explicit algorithm*:

5.3 $$ \textstyle\begin{cases} w_{n}=\alpha_{n}\gamma Vx_{n}+(I-\alpha_{n}\mu G)T_{r_{n}}{{\varLambda }}^{N}_{n}R_{n}x_{n}, \\ x_{n+1}=P_{C}[(I-\beta_{n}A)T_{r_{n}}{{\varLambda }}^{N}_{n}R_{n}x_{n}+ \beta_{n}w_{n}],\quad \forall n\geq 1, \end{cases} $$

*where* ${\varLambda }^{i}_{n}=T^{({\varTheta }_{i},\varphi_{i})}_{r _{i, n}}(I-r_{i, n}\nabla f_{i})T^{({\varTheta }_{i-1}, \varphi_{i-1})} _{r_{i-1,n}}(I-r_{i-1,n}\nabla f_{i-1})\cdots T^{({\varTheta }_{1}, \varphi_{1})}_{r_{1, n}}(I-r_{1,n}\nabla f_{1})$ *and* ${\varLambda } ^{0}_{n}=I$. *Assume that* $\{\alpha_{n}\}$, $\{\beta_{n}\}$, $\{r_{n}\}$, $\{\lambda_{n}\}$, $\{\nu_{n}\}$, *and* $\{r_{i, n}\}^{N}_{i=1}$ *satisfy conditions* (C1)*–*(C7) *in Theorem *3.3. *Then* $\{x_{n}\}$ *converges strongly to* $\widetilde{x}\in {{\varOmega }}:=\bigcap^{N}_{i=1}{\operatorname {MEP}}( {\varTheta }_{i}, \varphi_{i})\cap \bigcap^{N}_{i=1}\varPhi_{f_{i}} \cap \operatorname {GSVI}(C,F_{1},F_{2})\cap \operatorname {Fix}(T)$, *which is the unique solution of VI* (3.2).

### Proof

By using Lemma 5.1 and Theorem 3.3, we obtain the desired conclusion directly. □

## Conclusions

We introduced and analyzed one general implicit iterative scheme and another general explicit iterative scheme for finding a solution of a general system of variational inequalities (GSVI) with the constraints of finitely many generalized mixed equilibrium problems and a fixed point problem of a continuous pseudocontractive mapping in a Hilbert space. Moreover, we established strong convergence of the proposed implicit and explicit iterative schemes to a solution of the GSVI, which is the unique solution of a certain variational inequality. Our Theorems 3.1–3.3 not only improve and develop the main results of [1] and [12] but also improve and develop Theorems 3.1 and 3.2 of [9], Theorems 3.1 and 3.2 of [10], and Proposition 3.1, Theorems 3.2 and 3.5 of [11].
